# CMKLR1 senses chemerin/resolvin E1 to control adipose thermogenesis and modulate metabolic homeostasis

**DOI:** 10.1016/j.fmre.2022.06.014

**Published:** 2022-07-04

**Authors:** Zewei Zhao, Siqi Liu, Bingxiu Qian, Lin Zhou, Jianglin Shi, Junxi Liu, Lin Xu, Zhonghan Yang

**Affiliations:** a Department of Biochemistry, Molecular Cancer Research Center, School of Medicine, Sun Yat-sen University; Shenzhen, Guangdong 518107, China; b Department of Biochemistry, Zhongshan School of Medicine, Sun Yat-sen University; Guangzhou, Guangdong 510080, China; c School of Public Health, Sun Yat-sen University, Guangzhou, Guangdong 510080, China

**Keywords:** Obesity, Insulin resistance, Thermogenesis, CMKLR1, Resolvin E1, Chemerin

## Abstract

Induction of beige fat for thermogenesis is a potential therapy to improve homeostasis against obesity. β3-adrenoceptor (β3-AR), a type of G protein-coupled receptor (GPCR), is believed to mediate the thermogenesis of brown fat in mice. However, β3-AR has low expression in human adipose tissue, precluding its activation as a standalone clinical modality. This study aimed at identifying a potential GPCR target to induce beige fat. We found that chemerin chemokine-like receptor 1 (CMKLR1), one of the novel GPCRs, mediated the development of beige fat via its two ligands, chemerin and resolvin E1 (RvE1). The RvE1 levels were decreased in the obese mice, and RvE1 treatment led to a substantial improvement in obese features and augmented beige fat markers. Inversely, despite sharing the same receptor as RvE1, the chemerin levels were increased in obesogenic conditions, and chemerin treatment led to an augmented obese phenotype and a decline of beige fat markers. Moreover, RvE1 and chemerin induced or restrained the development of beige fat, respectively, via the mechanistic target of rapamycin complex 1 (mTORC1) signaling pathway. We further showed that RvE1 and chemerin regulated mTORC1 signaling differentially by forming hydrogen bonds with different binding sites of CMKLR1. In conclusion, our study showed that RvE1 and chemerin affected metabolic homeostasis differentially, suggesting that selectively modulating CMKLR1 may be a potential therapeutic target for restoring metabolic homeostasis.

## Graphical abstract

**Figure:**
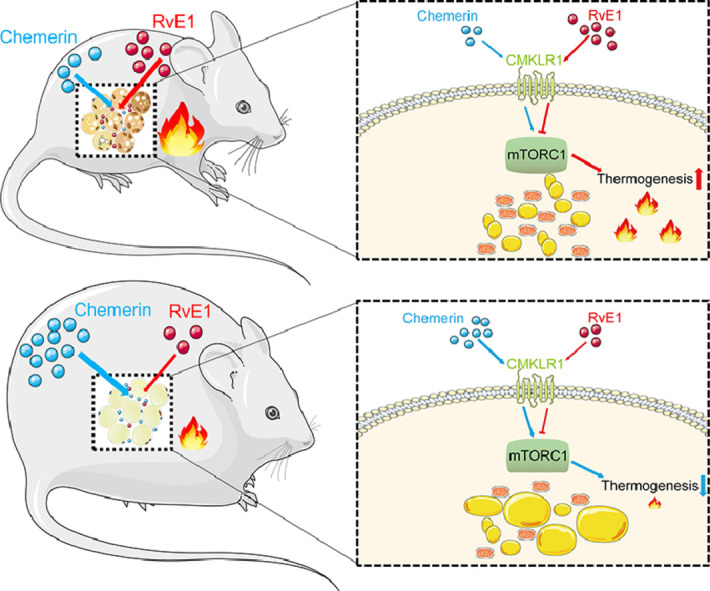

## 1 Introduction

The prevalence of obesity has nearly tripled worldwide in the past several decades. Nowadays, there is a consensus that obesity has become a global epidemic, with a massive economic burden attributable to its treatment [1,2]. As of 2019, the total number of overweight or obese adults worldwide exceeded 1.9 billion [3]. Obesity leads to higher risks of various metabolic diseases such as atherosclerosis, hypertension, insulin resistance and diabetes [4], [5], [6]. Recruitment and activation of beige/brown adipocytes have been shown to be potential therapeutic targets for the treatment of obesity and related metabolic diseases [7,8]. G protein-coupled receptors (GPCRs) are the most intensively studied drug targets, with about 34% of drugs approved by the US Food and Drug Administration (FDA) targeting at GPCRs [9]. β3-adrenergic receptor (β3-AR) is a GPCR believed to mediate brown fat thermogenesis in mice. However, its low expression in human adipose tissue, along with the cardiovascular risks associated with use of sympathomimetic drugs, probably precludes β3-AR activation from being explored as a standalone clinical modality [10,11]. Therefore, it is urgent to identify alternative GPCR targets to enable more effective therapies to be designed.

Chemerin is a chemokine that functions via binding to its receptor–CMKLR1, with the highest expression level in liver and white adipose tissue and moderate expression in brown adipose tissue [12], [13], [14]. Previous studies showed that chemerin may play a role in linking obesity and the associated comorbidities [15], [16], [17], but the underlying mechanism and its direct effects on the development of beige adipocytes remain unclear.

Resolvin E1 (RvE1) was firstly identified during the process of using peritoneal exudates for the resolution of inflammatory responses [18], [19], [20]. It has been subsequently found that weight loss increased the RvE1 levels in human neutrophils [21], suggesting that RvE1 may play an important role in the development of obesity. However, whether RvE1 is involved in the biogenesis of beige fat and thus induces weight loss and improves metabolic homeostasis is yet to be explored.

RvE1 preferentially binds to chemerin chemokine-like receptor 1 (ChemR23) [22]. ChemR23, also named CMKLR1-the receptor for chemerin, is expressed abundantly in macrophages and adipocytes [23,24]. Whether and how RvE1 and chemerin are involved in the development of beige adipocytes are still unclear. This study was aimed at investigating (1) the expressing effects of obesity on serum RvE1 and chemerin levels, (2) the browning effects of RvE1 and chemerin on different adipose tissues, (3) the effects of RvE1 and chemerin on metabolic homeostasis, and (4) the mechanisms underlying how RvE1 and chemerin differentially regulate the development of beige adipocyte.

## 2 Material and methods

### 2.1 Mice

Wild-type (WT) C57BL/6J mice were purchased from the center of laboratory animal of Sun Yat-sen University. Uncoupling protein 1 (UCP1) knockout (*Ucp1*^−/−^) mice were purchased from the model animal research center of Nanjing University. All mice were maintained under 12 h light-dark cycles with a designed environmental temperature (21 ± 1 °C) in Sun Yat-sen University Laboratory Animal Center. Eight-week-old C57BL/6J male mice were fed with a normal chow diet (NCD) or a high fat diet (HFD, 60% kcal) for 12 weeks to render mice obese. Except for HFD-fed mice, eight-week-old male mice were used in all experiments. For RvE1 and chemerin-regulating beiging experiments in NCD-fed, HFD-fed mice and *Ucp1*^−/−^mice, RvE1 (1µg/mouse, R144690, TRC) and vehicle (alcohol) or chemerin (10 ng/g, 50024-M08H, Sino Biological) and vehicle (bovine serum albumin, BSA) were injected intraperitoneally (i.p.) once daily for 14 days. After RvE1/chemerin-regulating beiging in NCD-fed mice or HFD-fed mice, glucose (1 g/kg for HFD-fed mice and 2 g/kg for NCD-fed mice, Sigma) or insulin (1 U/kg for HFD-fed mice and 0.5 U/kg for NCD-fed mice, Sigma) were injected i.p. at designed 0 min time points to perform intraperitoneal glucose or insulin tolerance tests in overnight-fasted mice. After injection, blood glucose concentration was measured using a OneTouch Ultra Glucometer (Johnson) at designed time points. Finally, the animals were euthanized and then tissue samples were collected. Cohorts of ≥ 4 mice per genotype or treatment were assembled for all in vivo studies. All in vivo studies were repeated 2–3 separated times. All procedures related to animal feeding, treatment and welfare were conducted at Sun Yat-sen University Laboratory Animal Center.

### 2.2 Metabolic rate and physical activity

Oxygen consumption and physical activity were determined for WT mice at 8 weeks of age using Comprehensive Lab Animal Monitoring System (CLAMS, Columbus Instruments) according to the manufacturer's instructions. RvE1 (1µg/mouse, R144690, TRC) and vehicle (alcohol) or chemerin (10 ng/g, 50024-M08H, Sino Biological) and vehicle (BSA) were injected i.p., respectively, once daily for 14 days. The animals were acclimated to the system for 20–24 h, and measurement of oxygen (O_2_) consumption and carbon dioxide (CO_2_) emission was performed during the next 24 h. The mice were maintained at 24 °C under a 12 h light/dark cycle. Food and water were available ad libitum. Voluntary activity was derived from the x-axis beam breaks monitored every 15 min. Metabolic cage results were analyzed by performing Analysis of Covariance (ANCOVA) through the Mouse Metabolite Phenotyping Center (http://www.mmpc.org/shared/regression.aspx).

### 2.3 Stromal vascular fraction (SVF) isolation

SVF from inguinal white adipose tissue (iWAT) of WT male mice at 4 weeks of age were washed with PBS, minced and digested with 0.1% type Ⅱ collagenase (Sigma) in Dulbecco's modified eagle medium (DMEM) containing 3% BSA and 25 µg/ml DNase Ι (Roche) for 30 min at 37 °C. During the digestion, the mixed solution was shaken by a hand every 5 min. The mixed solution was filtered through a 70 µm cell strainer (Falcon) and then centrifuged at 500 g for 5 min at 4 °C. The floating adipocytes were removed, and the pellets containing SVF were resuspended in red blood cell lysis buffer (Sigma) for 5 min at 37 °C. Cells were centrifuged at 500 g for 10 min at 4 °C and the pellets were re-suspended in DMEM medium containing 10% fetal bovine serum (FBS).

### 2.4 Cell culture

3T3-L1 and Hela cell lines were purchased from the Cell Bank of the Chinese Academy of Sciences in Shanghai. Confluent pre-adipocytes (3T3-L1 and SVF) were induced into mature adipocytes with 0.5 mM isobutyl methylxanthine (IBMX), 1 µM dexamethasone (Dex) and 5 µg/ml insulin in DMEM containing 10% FBS for 3 days, then treated with DMEM containing 5 µg/ml insulin and 10% FBS for 6 days and cultured with DMEM containing 10% FBS for 2 days. Various doses of RvE1 (R144690, TRC)/chemerin (50024-M08H, Sino Biological), CL-316,243 (CL, 1 µM) or Mirabegron (Mi, 10 µM) were added to mature adipocytes for 24 h. The Hela cell line was maintained in DMEM supplemented with 10% FBS for signal transduction studies. 100 ng/ml RvE1 or 1 µM chemerin-9 (the nonapeptide 149-YFPGQFAFS-157, corresponding to the C terminus of processed chemerin that retains most of the activity of the full-size protein as an agonist to CMKLR1 [25]) was added to Hela cell for 24 h.

### 2.5 Clustered regularly interspaced short palindromic repeats (CRISPR)-mediated gene knockout

The sequences targeting *Cmklr1* were *Cmklr1* gRNA1 (5′-TCTGGCCTCGTGTTCCTACCTGG-3′) and *Cmklr1* gRNA2 (5′-GACTGGATTCGAATCGCAGTGGG-3′). The Cas9 lentivirus vector and gRNA1/2 lentivirus vector were purchased from Cyagen Biosciences and transduced to 3T3-L1 cells. The transduced cells were screened by flow cytometry to obtain the *Cmklr1* knockout 3T3-L1 cells.

### 2.6 Construction of point mutant plasmid

The point mutant plasmid of human CMKLR1 was purchased from Shanghai Generay Biotechnology.

### 2.7 Temperature measurements

The body temperature was measured at 9:00 using a rectal probe connected to a digital thermometer (BAT-12 Microprobe-Thermometer; Physitemp; NJ, USA). The body temperature of each mouse was measured three times, and average body temperature was calculated and recorded.

### 2.8 Real-time polymerase chain reaction (PCR)

Total RNA from tissue or cells was extracted with Trizol reagent (Invitrogen). RNA concentration was measured by a NanoDrop spectrometer. 1000 ng total RNA was reverse transcribed into cDNA by Prime Script® RT reagent Kit Perfect Real Time kit (TaKaRa). Real-time PCR analysis using SYBR-Green fluorescent dye (Biorad) was performed with a Biorad CFX 96. Primers used for real-time PCR were listed in Table 1.

**Table 1:** **Primer sequences used for qPCR**.

**Gene**	**Forward Primer**	**Reverse Primer**
*Pgc-1α*	TATGGAGTGACATAGAGTGTGCT	CCACTTCAATCCACCCAGAAAG
*Pparα*	AGGCTGTAAGGGCTTCTTTCG	GGCATTTGTTCCGGTTCTTC
*Ampk*	GGTGTTATCCTGTATGCCCTTCT	TGTCTTTGATAGTTGCTCGCTTC
*Cmklr1*	ATGGAGTACGACGCTTACAACG	GGTGGCGATGACAATCACCA
*Ucp1*	GGCCTCTACGACTCAGTCCA	TAAGCCGGCTGAGATCTTGT
*Cidea*	TGCTCTTCTGTATCGCCCAGT	GCCGTGTTAAGGAATCTGCTG
*Cox8b*	TGTGGGGATCTCAGCCATAGT	AGTGGGCTAAGACCCATCCTG
*Prdm16*	TGACCATACCCGGAGGCATA	GGTACCCTGGCTTTGGACTC
*Chemerin*	ATGGAGTACGACGCTTACAACG	CTTGCTTCAGAATTGGGCAGT
*Cyto c*	CCAAATCTCCACGGTCTGTTC	ATCAGGGTATCCTCTCCCCAG
*Atp-syn*	GGTTCATCCTGCCAGAGACTA	AATCCCTCATCGAACTGGACG
*β-Actin*	TGTCCACCTTCCAGCAGATGT	AGCTCAGTAACAGTCCGCCTAGA
*mito-16s*	CCGCAAGGGAAAGATGAAAGAC	TCGTTTGGTTTCGGGGTTTC

### 2.9 Histology and immunohistochemistry

Epididymal, subcutaneous white adipose tissue and interscapular brown adipose tissue were fixed in 4% paraformaldehyde. Tissues were embedded with paraffin and sectioned by microtome. The slides were stained with hematoxylin and eosin (HE) using a standard protocol. For UCP1 immunohistochemistry, slides of various tissue were blocked with goat serum for 1 h. Subsequently, the slides were incubated with anti-UCP1 (1:1,000; ab10983, Abcam) overnight at 4°C followed by detection with the EnVision Detection Systems (K5007, Dako). Hematoxylin (ZSGB-BIO) was used as counterstain.

### 2.10 Western-blot

Tissues and cells were lysed in RIPA buffer (CST) supplemented with 1 mM PMSF (Beyotime). The protein concentration was measured by the KeyGen protein assay kit (KeyGen), and total cellular protein (30 µg) was subject to Western-blot analysis. The protein transferred to the PVDF membrane (Millipore) was probed with primary antibodies specific for HSP90 (1:1,000; C45G5, CST), β-actin (1:5,000; A5441, Sigma), GAPDH (1:5,000; G8795, Sigma), UCP1 (1:1,000 for epididymal white adipose tissue (eWAT), iWAT and cells or 1:1,0000 for brown adipose tissue (BAT); ab10983, Abcam), CMKLR1 (1:500; sc-398769, Santa Cruz), p-S6K (1:1,000; 9205, CST) and S6K (1:1,000; 2708, CST) overnight at 4 °C. After being incubated with goat anti-rabbit IgG/HRP (1:1,000; PI1000, Vector Laboratories) secondary antibody, proteins were detected with chemiluminescence using Immobilon Western HRP Substrate (Merck Millipore) on Image quant LAS 4000-mini (GE Healthcare). The ImageJ software was used for gray scanning. For all Western-blots, each lane represented an independent sample and all experiments were replicated 2 to 3 times.

### 2.11 Enzyme-linked immunosorbent assay (ELISA)

Mouse chemerin level was detected using a sensitive ELISA kit (E-EL-M0015c; Elabscience). Mouse RvE1 level was detected using a sensitive ELISA kit (MM-44695M2; MEIMIAN). Human chemerin level was detected using a sensitive ELISA kit (MS-1763H2; Maisha). Human RvE1 level was detected using a sensitive ELISA kit (MM-14657H2; MEIMIAN). Mouse Insulin level was detected using a sensitive ELISA kit (EW0351Mo; ELGBIO). All measurements were performed using the manufacture's protocol. Plasma, liver and adipose tissues were rinsed and homogenized in PBS. The plasma was generated from peripheral blood of mice by centrifuged at 3,000 r/min at 4 °C for 20 min. The homogenates were centrifuged for 5 min at 10,000 rpm for 10 min at 4 °C and then the supernate was assayed immediately. All samples were normalized to total tissue protein concentration.

### 2.12 Oil Red O staining

The slices were fixed with 4% paraformaldehyde. After fixation, lipid droplets of tissue slices were stained by the Oil Red O prepared in 2-propanol (0.5%) as the stock solution, and the working solution was freshly diluted with distilled deionized water. The slices were incubated with Oil Red O working solution (60 ml stock solution mixed with 40 ml deionized water) for 15 min and were stained with hematoxylin for 30 s. All the slices were observed under a light contrast microscope.

### 2.13 Measurement of triacylglycerol (TG) and adenosine triphosphate (ATP)

The triacylglycerol in tissues and plasma was measured by using EnzyChromTM Triglyceride Assay Kit (Cat# ETGA-200) according to the manufacturer's instructions. The ATP produced by 3T3-L1 was measured with an ATP detection kit (Beyotime Biotechnology, China, #S0026) according to the manufacturer's instructions.

### 2.14 Mito-tracker staining

3T3-L1 were incubated with 100 nM Mito-Tracker Red CMXRos (Beyotime Biotechnology, China, #C1049) for 30 min according to manufacturer's instructions. Then cells were washed with PBS and visualized under the confocal microscope.

### 2.15 Transmission electron microscopy

BAT and iWAT sections were fixed in 2% (vol/vol) glutaraldehyde in 100mM phosphate buffer, pH 7.2 for 12 h at 4 °C. The sections were then post-fixed in 1% osmium tetroxide, dehydrated in ascending gradations of ethanol and embedded in fresh epoxy resin 618. Ultra-thin sections (60-80 nm) were cut and stained with lead citrate before being examined on the FEI-Tecnai G2 Spirit Twin transmission electron microscope.

### 2.16 Differential expression analysis

The R package Linear Models for Microarray Data (limma) and Empirical Analysis of Digital Gene Expression Data in R (edgeR) was used to analyze differential RNA-Sequencing expression. For screening potential G-protein-coupled receptors regulating the biogenesis of beige fat, edgeR was applied in the GSE118849 dataset to screen out differentially expressed genes (DEGs). To find the DEGs between *Rarres2* knockout and WT groups, limma was applied in the GSE133978 dataset to screen out DEGs. Genes with the cutoff criteria of |logFC| ≥ 1.0 and *p* < 0.05 were regarded as DEGs. The DEGs of the GSE118849 and GSE133978 datasets were visualized as a volcano plot by using the R package ggplot2.

### 2.17 Functional annotation for genes of interest

To explore Gene Ontology (GO) of selected genes, Metascape was used to explore the functions among DEGs, with a cutoff criterion of *p* < 0.05. GO annotation that contains the biological process (BP) subontology, which can identify the biological properties of genes and gene sets for all organisms.

### 2.18 Gene set enrichment analysis (GSEA)

GSEA was performed to detect a significant difference in the set of genes expressed between the *Rarres2* knockout and WT groups in the enrichment of the KEGG collection.

### 2.19 Docking calculations, molecular dynamics simulations and binding free-energy calculations

All docking calculations were performed with the AutoDock Vina program. The crystal structure of human CMKLR1 was used as a reference point. The PDB structure of 6os0.1.A was used as a template for the predication of human CMKLR1. The PDB structure of mouse CMKLR1 was predicated by AlphaFold [26,27]. All docking calculations were performed with the AutoDock Vina program.

### 2.20 Availability of data and materials

The transcriptomic dataset analyzed in this study can be accessed on the GEO repository under accession numbers GSE133978 and GSE118849. The chemical structure of RvE1/chemerin-9/α-NETA, the crystal structure of human CMKLR1 and mouse CMKLR1 analyzed in this study, and the computational models of RvE1/chemerin-9/α-NETA bound to human CMKLR1 and mouse CMKLR1 have been deposited into the Zenodo (https://doi.org/10.5281/zenodo.6431583). All other data associated with this paper can be found in the main text or the Supplementary Materials.

### 2.21 Statistical analysis

All data are presented as mean ± *SEM*. Student's t-test was used to compare two groups. One-way analysis of variance (ANOVA) or Two-way ANOVA was applied to compare more than two different groups on GraphPad Prism 9.0 software. For each parameter of all data presented, NS (No Significance), *p < 0.05, ** *p <* 0.01, *** *p <* 0.001 and **** *p <* 0.0001. *p <* 0.05 is considered significant.

## 3 Results

### 3.1 Transcriptomic analysis identifies CMKLR1 as a potential GPCR regulating the development of beige fat

**Fig. 1:**
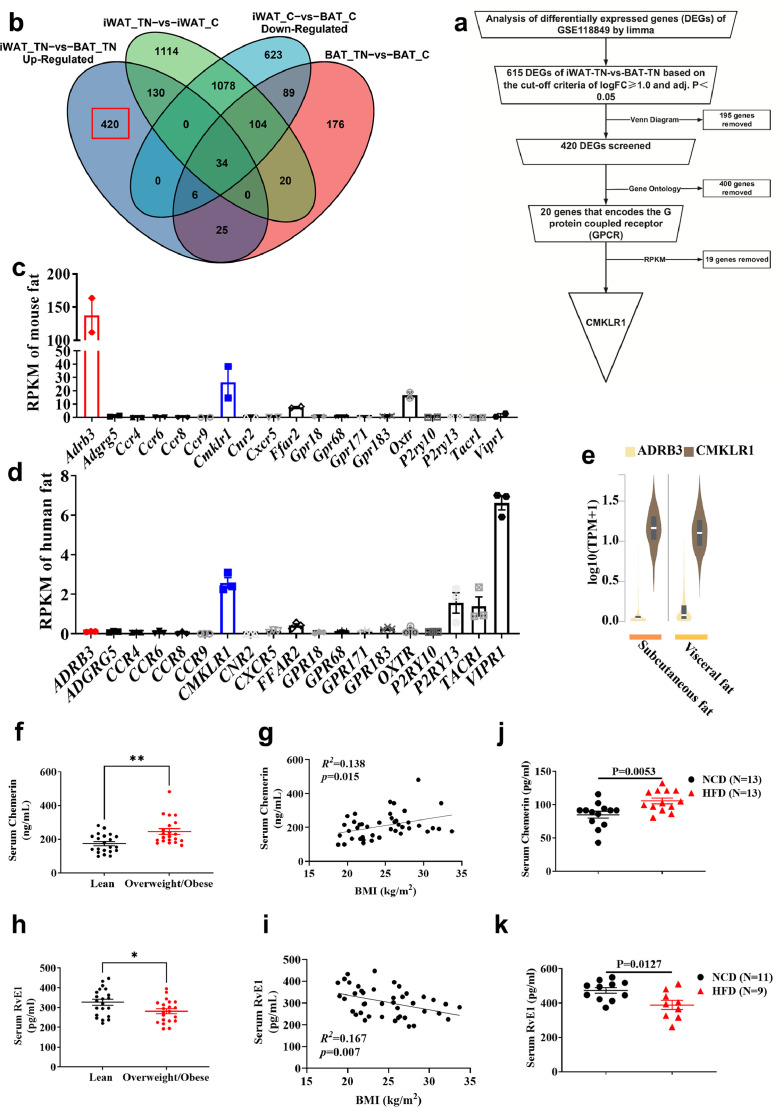
**CMKLR1 signaling is associated with fat thermogenesis and fatness.** (a–e) CMKLR1 screening as a potential GPCR associated with fat thermogenesis via transcriptomic analysis. Brown adipose tissue and subcutaneous WAT were dissected from mice that were treated in thermo-neutral (30 °C) and cold (4 °C) temperatures for 72 h. A total of 12 samples with three replicates for each condition were evaluated. (a) Flowchart of screening. (b) Four groups DEGs from transcriptome were analyzed by using a Venn diagram. iWAT-TN-vs-BAT-TN Up-regulated: the up-regulated DEGs in the thermal-neutral iWAT group compared with thermal-neutral BAT group. iWAT_TN-vs-iWAT_C: the DEGs between the thermal-neutral iWAT group and cold temperature iWAT group. iWAT_C-vs-BAT_C Down-Regulated: the down-regulated DEGs in the cold temperature iWAT group compared with cold temperature BAT group. BAT_TN-vs-BAT_C: the DEGs between thermal-neutral BAT group and cold temperature BAT group. (c, d) The RPKM of 19 GPCR genes in mouse fat (c) from Mouse ENCODE transcriptome data (PRJNA66167) and human fat (d) from HPA RNA-seq normal tissues (PRJEB4337). (e) The TPM of ADRB3 and CMKLR1 in human fat from GTEx database. (f, h) The level of chemerin (f) and RvE1 (h) in the serum of lean and overweight/obese people. Two ELISA kits were used to measure the level of chemerin and RvE1. Lean (BMI = 18.5∼23.9) *n* = 21, Overweight/Obese (BMI > 25.5) *n* = 21. (g) Correlation between the serum chemerin level and body mass index (BMI). (i) Correlation between the serum RvE1 level and body mass index (BMI). (j–k) The serum level of chemerin (j) and RvE1 (k) was measured in the serum of mice fed either a NCD or HFD. Two ELISA kits were used to measure the level of mouse chemerin and RvE1. TN, thermo-neutral; C, cold temperature; iWAT, inguinal white adipose tissue; BAT, brown adipose tissue; RPKM, Reads Per Kilobase per Million mapped reads; TPM, Transcripts Per Kilobase Million; GPCR, G-protein-coupled receptor; BMI, body mass index; NCD, normal chow diet; HFD, high-fat diet; RvE1, Resolvin E1. All data are presented as mean ± *SEM*. Statistical significance was determined by unpaired two-tailed student's t-test (f, h and j, k) or simple linear regression (g, i).

We identified CMKLR1 as a potential GPCR target via transcriptomic analysis (Fig. 1a) that regulates the development of beige fat via two different ligands—chemerin and RvE1. To find innovative GPCR regulating the biogenesis of beige fat, we performed differential gene expression analysis (Fig. S1a–d) and Venn diagram analysis (Fig. 1b) on the GSE118849 dataset from the Gene Expression Omnibus (GEO) database. The GSE118849 dataset contains brown adipose tissue (BAT) and inguinal white adipose tissue (iWAT) dissected from mice that were treated in thermo-neutral (30 °C) and cold temperature (4 °C) for 72 h. The differentially expressed genes (DEGs) between thermal-neutral iWAT group and cold temperature iWAT (Fig. S1b), DEGs between thermal-neutral BAT group and cold temperature BAT group (Fig. S1d) and DEGs, which were down-expressed in WAT compared with BAT under cold stimulation (Fig. S1c, red), were excluded in the analysis to find GPCRs that is highly expressed in white adipose tissue (WAT) compared with BAT. A total of 420 DEGs that were highly expressed in WAT compared with BAT under thermal neutral condition (Fig. S1a, blue) were screened out (Fig. 1b, marked in red box). We further annotated 420 DEGs and found that 20 of them were involved in encoding GPCR (Table 2). Among these 20 genes, Reads Per Kilobase per Million mapped reads (RPKM) data corresponding to FFAR1 and GPR174 genes were lacking and not included in subsequent analysis.

**Table 2:** **Gene annotation for screened genes.** The 420 screened genes were annotated by David database and 20 of these genes were identified as G-protein coupled receptor encoding gene.

**Gene ID**	**Gene Name**	**GO Annotation**
382045	**ADGRG5**	GO0004930: G-protein coupled receptor activity
12773	**CCR4**	GO0004930: G-protein coupled receptor activity
12458	**CCR6**	GO0004930: G-protein coupled receptor activity
12776	**CCR8**	GO0004930: G-protein coupled receptor activity
12769	**CCR9**	GO0004930: G-protein coupled receptor activity
14747	**CMKLR1**	GO0004930: G-protein coupled receptor activity
12802	**CNR2**	GO0004930: G-protein coupled receptor activity
12145	**CXCR5**	GO0004930: G-protein coupled receptor activity
233081	**FFAR1**	GO0004930: G-protein coupled receptor activity
233079	**FFAR2**	GO0004930: G-protein coupled receptor activity
229323	**GPR171**	GO0004930: G-protein coupled receptor activity
213439	**GPR174**	GO0004930: G-protein coupled receptor activity
110168	**GPR18**	GO0004930: G-protein coupled receptor activity
321019	**GPR183**	GO0004930: G-protein coupled receptor activity
238377	**GPR68**	GO0004930: G-protein coupled receptor activity
18430	**OXTR**	GO0004930: G-protein coupled receptor activity
78826	**P2RY10**	GO0004930: G-protein coupled receptor activity
74191	**P2RY13**	GO0004930: G-protein coupled receptor activity
21336	**TACR1**	GO0004930: G-protein coupled receptor activity
22354	**VIPR1**	GO0004930: G-protein coupled receptor activity

We further evaluated the expression levels of 18 remaining GPCRs and β3-AR in adipose tissue. The results showed that the expression of CMKLR1 was second only to that of β3-AR in mouse adipose tissue (Fig. 1c), while CMKLR1 was higher than that of β3-AR in human adipose tissue (Fig. 1d, 1e). Then we investigated how high-fat-diet regulated the transcription of *Cmklr1* and *Ucp1* (Uncoupling protein 1, a functional protein and marker of beige/brown fat). The results showed that HFD significantly inhibited the expression of *Cmklr1* and *Ucp1* in iWAT, epididymal white adipose tissue (eWAT) and BAT (Fig. S1e, 1f). Interestingly, *Cmklr1* and *Ucp1* showed opposite trends in the expression levels of these three adipose tissues under NCD feeding (Fig. S1e, 1f). These data suggest that CMKLR1 is a GPCR that is expressed highly in WAT and may participate in regulating adipogenesis and thermogenesis.

We then compared the level of two ligands of CMKLR1, RvE1 and chemerin, in the serum from humans that were lean or overweight/obese, and found that chemerin was increased (Figs. 1f, g and S1g) whereas RvE1 was decreased (Figs. 1h, i and S1g) in the serum from overweight/obese humans. Moreover, the ratio of serum chemerin level to RvE1 level was positively correlated with body mass index (BMI; Fig. S1h). Similar results were observed in mice. Chemerin was increased (Fig. 1j) while RvE1 was decreased (Fig. 1k) in the serum from mice fed the HFD as compared with that from mice fed NCD. Therefore, two ligands of CMKLR1 may correlate with the development of obesity.

To elucidate the intrinsic relationship between chemerin and the development of beige fat, we analyzed the RNA-sequencing dataset of cold stimulated inguinal fat of WT and *Rarres2*^−/−^ (the encoding gene of chemerin) mice from the GSE133978 dataset. A total of 48 genes were differentially expressed (Fig. S2a). The biological process (BP) of 48 genes was mainly enriched in carbohydrate metabolic process and adaptive thermogenesis (Fig. S2b). Selective β3-adrenoceptor agonist (CL-316,243 (hereby referred to as CL)) treatment led to obvious repression of chemerin in iWAT (Fig. S2c), eWAT (Fig. S2c) and serum (Fig. S2d), which was also verified by real-time quantitative PCR (Fig. S2e, S2f). Meanwhile, the expression of beige markers was up-regulated and the expression of chemerin was down-regulated in both iWAT (Fig. S2e and g) and eWAT (Fig. S2f, 2g) after CL treatment, as well as a higher expression of *Cmklr1* and *Chemerin* in iWAT and eWAT compared with BAT (Fig. S2h). Collectively, these results reveal that chemerin is negatively correlated with the biogenesis of beige fat.

### 3.2 RvE1 and chemerin regulate the biogenesis of beige adipocyte differentially in vitro

**Fig. 2:**
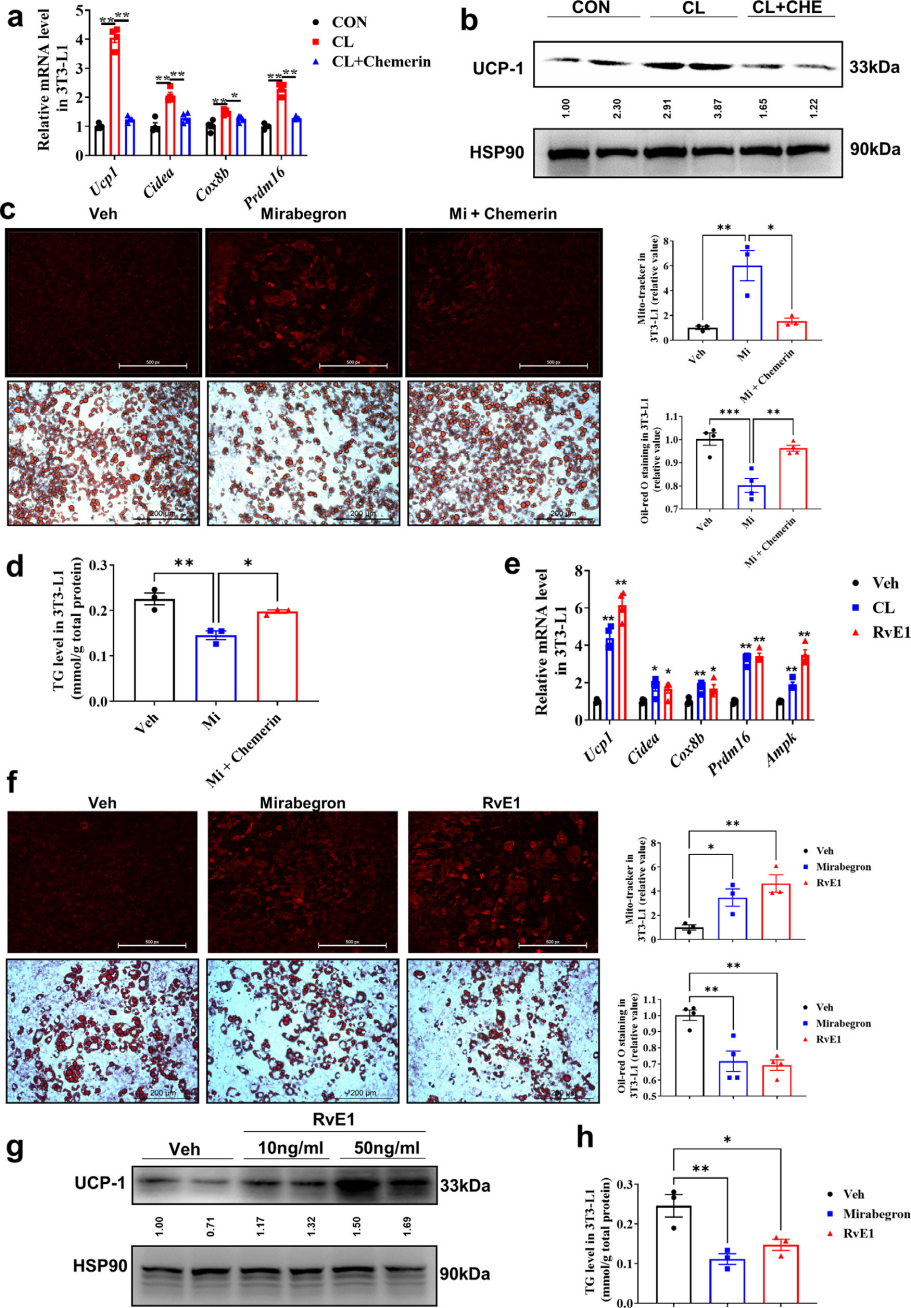
**Chemerin and RvE1 differentially regulated the biogenesis of beige adipocytes.** 3T3-L1 mature adipocytes were treated with 1 µM CL, 10 µM Mi, 1 µM CL with 100 ng/ml chemerin (CL+CHE), 10 µM Mi with 100 ng/ml chemerin (Mi+chemerin) or 100 ng/ml RvE1. (a, e) qPCR analysis of markers associated with biogenesis of beige adipocyte in 3T3-L1. (b, g) Western-blot analysis for the level of UCP1 protein in 3T3-L1. The ImageJ software was used for gray scanning. (c, f) Mito-Tracker red staining for mitochondria (top; scale bars, 500 µm), Oil Red O staining for lipid droplets (bottom; scale bars, 200 µm) and staining intensity analysis diagram (right). (d, h) The level of intracellular triglyceride. All data are presented as mean ± *SEM*. Statistical significance was determined by one-way ANOVA (a, c–h).

To clarify whether RvE1 and chemerin regulate the biogenesis of beige adipocytes, we transformed pre-adipocytes 3T3-L1 into mature adipocytes with the treatment of gradient drugs and the β3-adrenoceptor agonist (CL or Mirabegron (Mi)) used as positive control for induction of UCP1. The expression of beige fat marker genes was up-regulated by CL/Mi (Figs. 2a and S6a), but were down-regulated by chemerin. Western-blot and Mito-Tracker staining showed that the expression of UCP1 (Fig. 2b) and the number of mitochondria (Fig. 2c, top) were increased after CL/Mi treatment, which could be reversed by chemerin. Oil red O staining and intracellular triglyceride assay of adipocytes showed a decrease in lipid droplets (Fig. 2c, bottom) and intracellular triglyceride level (Fig. 2d) after treatment with Mirabegron, which can be reversed by chemerin. After being treated with RvE1, the expression of beige related genes in 3T3-L1 was up-regulated (Figs. 2e and S6a), the production of ATP was enhanced (Fig. S6b) and Mito-Tracker staining showed an increase in the number of mitochondria (Fig. 2f, top). RvE1 increased the expression of UCP1 in a dose-dependent manner (Fig. 2g). In addition, there was a decrease in the lipid droplets (Fig. 2f, bottom) and intracellular triglyceride levels (Fig. 2h) after treatment with RvE1. These data indicate that RvE1 and chemerin can differentially regulate the biogenesis of beige adipocyte in vitro.

### 3.3 Chemerin suppresses adipose thermogenic program and impairs metabolic homeostasis dependent on UCP1

To evaluate the role of chemerin in the biogenesis of beige fat in vivo, mice fed the HFD for 12 weeks were injected with vehicle, CL or CL with chemerin for 5 days. We found that chemerin almost completely reversed the weight-loss and thermogenesis effects of CL (Fig. S3a, 3b). In addition, histological examination, RT-qPCR and Western-blot showed that CL treatment was sufficient to induce potent beige fat formation which could be obstructed by chemerin (Fig. S3c–h).

The metabolic effects of chemerin were further examined by administering chemerin to WT mice or *Ucp1* knockout (*Ucp1*^−/−^) mice fed the NCD. After 14 days of chemerin treatment, the weight of the WT mice increased remarkably, but not in *Ucp1*^−/−^ mice (3a), and the food intakes for both mice were not influenced (Fig. S4a). Serum triacylglycerol (TG) levels (Fig. S4b), liver weight ratio (Fig. S4c), liver TG levels (Fig. S4d) and iWAT TG levels (Fig. S4e, left) were significantly higher in chemerin-treated WT mice, but not *Ucp1*^−/−^ mice. TG levels of eWAT (Fig. S4e, right) tended to increase, but there was no statistically significant difference. Meanwhile, hematoxylin-eosin staining showed that chemerin induced adipose expansion in iWAT (Fig. S4f, top), eWAT (Fig. S4f, middle) and BAT (Fig. S4f, bottom) in WT but not *Ucp1*^−/−^ mice.

**Fig. 3:**
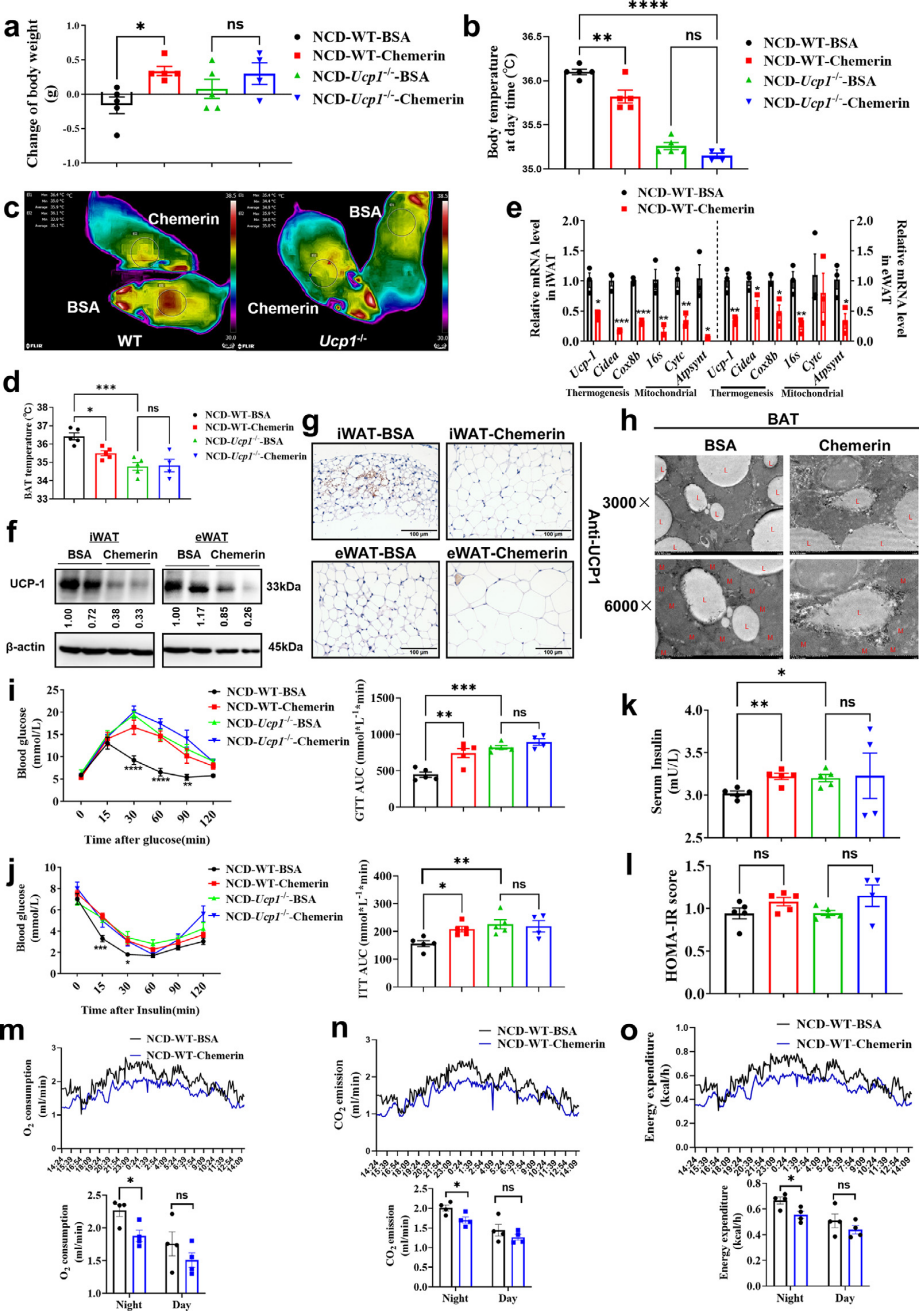
**Chemerin suppressed the adipose thermogenic program and impaired metabolic homeostasis in mice dependent on UCP1.** (a–k) C57BL/6J mice or *Ucp1* knockout mice fed with NCD for 8 weeks (*n* = 5 per treatment) were injected with BSA (10 ng/g/day) or chemerin (10ng/g/day) over 14 days. (a, b) Changes in body mass (a) and body temperature (b) in wild-type and *Ucp1*-knockout mice injected with vehicle or chemerin for 14 days. (c, d) Thermal image (c) and BAT temperature (d) in wild-type and *Ucp1*-knockout mice injected with vehicle or chemerin for 14 days. (e) qPCR analysis of markers associated with thermogenesis and mitochondrial in iWAT and eWAT from different treatment mice. (f) Western-blot analysis for the level of UCP1 protein in iWAT and eWAT from differently treated mice. The ImageJ software was used for gray scanning. (g) Representative images of iWAT (top) and eWAT (bottom) stained with UCP1. Scale bars, 100 µm. (h) Transmission electron microscope photograph of BAT treated with BSA or chemerin. The red ‘L’ marks represent lipid droplets and the red ‘M’ marks represent mitochondria. (i) Glucose tolerance test (GTT) was conducted by intraperitoneal injection of glucose (2 g/kg) and measurement of blood glucose concentration with a OneTouch Ultra Glucometer at designed time points in overnight-fasted mice. (j) Insulin tolerance test (ITT) was done by intraperitoneal injection of insulin (0.5 U/kg) and measurement of blood glucose concentration by a OneTouch Ultra Glucometer at designed time points in 12 hr-fasted mice. (k–l) The fasting serum insulin (k) and HOMA-IR (l) in mice injected with BSA or chemerin for 14 days. HOMA-IR = Fasting glucose level (mmol/L) × Fasting insulin level (mIU/L)/22.5. (m–o) The O_2_ consumption (m), CO_2_ production (n) and the calorie consumption (o) of C57BL/6J mice fed with NCD for 8 weeks (*n* = 4 per treatment) injected with BSA (10 ng/g/day) or chemerin (10 ng/g/day) over 14 days. All data are presented as mean ± *SEM*. Statistical significance was determined by one-way ANOVA (a, b, d and k, l) or two-way ANOVA (i, j). Metabolic cage results were analyzed by performing ANCOVA (M-O) through the Mouse Metabolite Phenotyping Center (http://www.mmpc.org/shared/regression.aspx).

Strikingly, chemerin treatment was sufficient to decrease body temperature (Fig. 3b) and BAT temperature (Fig. 3c, 3d) in WT mice, with no significant effect in *Ucp1*^−/−^ mice (Fig. 3b–d). Chemerin significantly decreased the expression of beige fat-related genes in both iWAT (Fig. 3e, left) and eWAT (Fig. 3e, right). Moreover, Western-blot (Figs. 3f and S4g) and Immunohistochemical staining (Figs. 3g and S4h) of UCP1 identified similar effects. Furthermore, we found that after treatment with chemerin, the BAT expressed expanded lipid droplets and lessened mitochondria (Fig. 3h). Thus, these data suggest that chemerin serves as a negative regulatory factor in the biogenesis of beige fat and the activation of BAT.

We found that chemerin-treated mice had dramatically impaired whole-body glycemic homeostasis (Fig. 3i) and insulin sensitivity (Fig. 3j), which were UCP1 dependent. Moreover, the fasting serum insulin level was increased after chemerin treatment or *Ucp1* gene ablation (Fig. 3k), even though the homeostasis model assessment of insulin resistance (HOMA-IR) showed a modest increase without a statistical difference (Fig. 3l). In addition, we measured oxygen consumption (Fig. 3m), carbon dioxide production (Fig. 3n), and calorie consumption (Fig. 3o) and found that the energy expenditure of chemerin-treated mice was significantly weakened. Thus, these results demonstrated that chemerin inhibits the biogenesis of beige fat and deteriorates metabolic homeostasis in vivo, which is UCP1 dependent.

### 3.4 RvE1 activates the adipose thermogenic program and counteracts metabolic disease dependent on UCP1

**Fig. 4:**
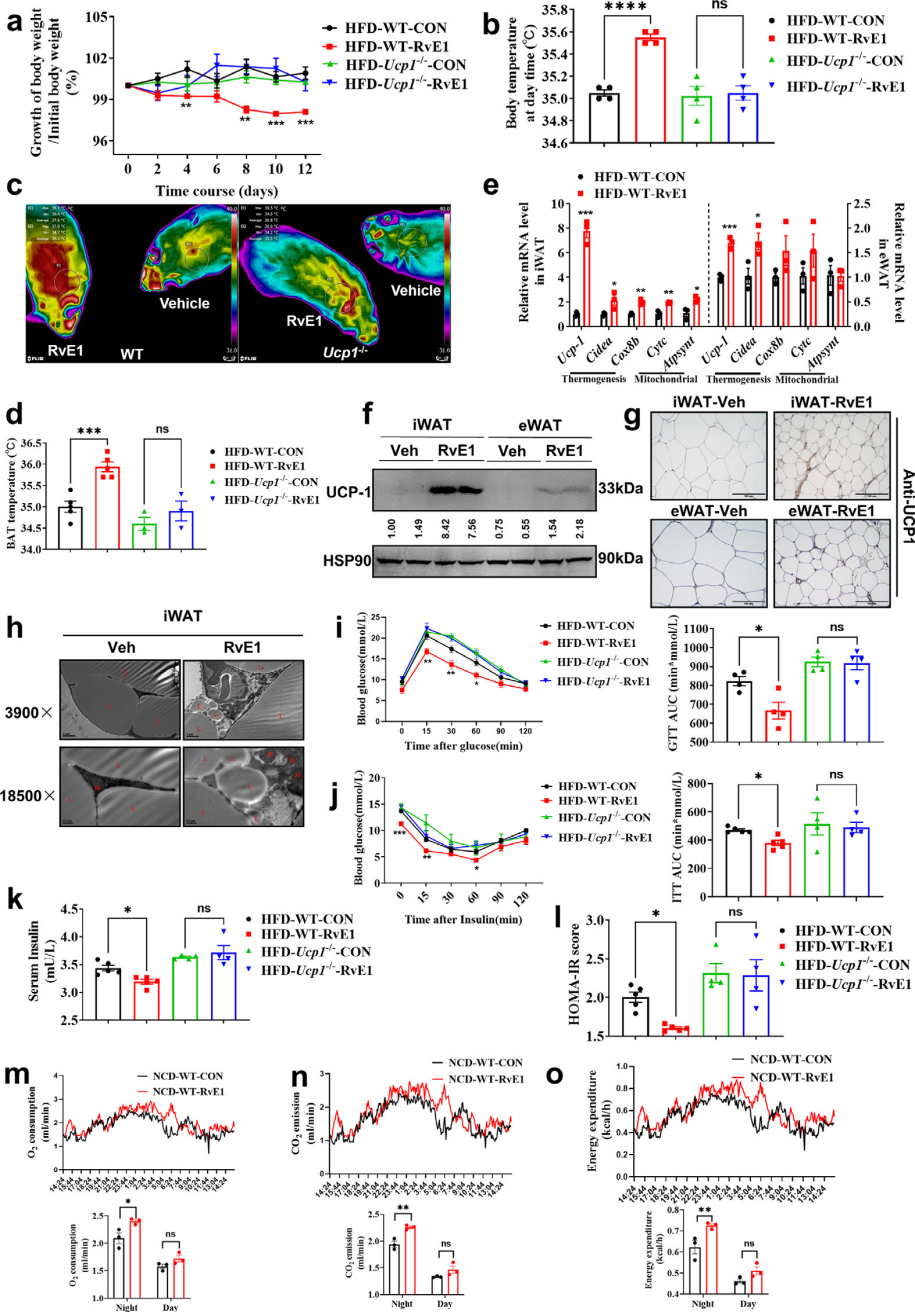
**RvE1 activated the adipose thermogenic program and facilitated metabolic homeostasis in mice with diet-induced obesity (DIO) dependent on UCP1.** (a–l) C57BL/6J mice fed with HFD for 12 weeks (*n* = 5 per treatment) were injected with vehicle or RvE1 (1 µg/mouse) over 14 days. (a, b) Changes in body mass (a) and body temperature (b) in mice fed the HFD to induce obesity were injected with vehicle or RvE1 for 14 days. (c, d) Thermal image (c) and BAT temperature (d) in wild-type and *Ucp1*-knockout mice injected with vehicle or RvE1 for 14 days. (e) qPCR analysis of markers associated with the biogenesis of beige fat and thermogenesis in iWAT (left) and eWAT (right) from different treated mice. (f) Western-blot analysis for level of UCP1 protein in iWAT and eWAT from different treated mice. The ImageJ software was used for gray scanning. (g) Representative images of iWAT and eWAT stained with UCP1. Scale bars, 100 µm. (h) Transmission electron microscope photograph of iWAT treated with vehicle or RvE1. The red ‘L’ marks represent lipid droplets and the red ‘M’ marks represent mitochondria. (i) Glucose tolerance test (GTT) was conducted by intraperitoneal injection of glucose (1 g/kg) and measurement of blood glucose concentration with a OneTouch Ultra Glucometer at designed time points in overnight-fasted mice. (j) Insulin tolerance test (ITT) was done by intraperitoneal injection with insulin (1 U/kg) and measurement of blood glucose concentration by a OneTouch Ultra Glucometer at designed time points in 12 h-fasted mice. (k, l) The fasting serum insulin (k) and HOMA-IR (l) in mice injected with vehicle or RvE1 for 14 days. HOMA-IR = Fasting glucose level (mmol/L) × Fasting insulin level (mIU/L)/22.5. (m–o) The O_2_ consumption (m), CO_2_ production (n) and the calorie consumption (o) of C57BL/6J mice fed with NCD for 8 weeks (*n* = 3 per treatment) injected with Vehicle or RvE1 (1 µg/day) over 14 days. All data are presented as mean ± *SEM*. Statistical significance was determined by unpaired two-tailed Student's t-test (k, l), one-way ANOVA (b, d and k, l) or two-way ANOVA (a, i, j). Metabolic cage results were analyzed by performing ANCOVA (m–o) through the Mouse Metabolite Phenotyping Center (http://www.mmpc.org/shared/regression.aspx).

To identify whether RvE1 improves the metabolic homeostasis against obesity via inducing the biogenesis of beige fat, RvE1 injected i.p. into WT mice or *Ucp1*^−/−^ mice was found to alleviate the growth of body weight (Fig. 4a) during the HFD feeding despite maintaining comparable levels of food intake (Fig. S5a), which was dependent on UCP1. RvE1 decreased the serum TG levels (Fig. S5b), liver weight ratio (Fig. S5c), liver TG levels (Fig. S5d), iWAT TG levels (Fig. S5e) and BAT TG levels (Fig. S5g) and increased body temperature (Fig. 4b) and BAT temperature (Fig. 4c, 4d) in WT mice, which were significantly blunted in *Ucp1*^−/−^ mice. Meanwhile, RvE1 decreased the area of adipocytes in iWAT (Fig. S5h, top), eWAT (Fig. S5h, middle) and BAT (Fig. S5h, bottom), and this effect was absent in *Ucp1*^−/−^ mice.

Moreover, the thermogenic genes were elevated in iWAT (Fig. 4e, left) and eWAT (Fig. 4e, right). Western-blot (Figs. 4f and S5i) and Immunohistochemical staining of UCP1 (Figs. 4g and S5j) showed that the expression of UCP1 was increased dramatically in iWAT, eWAT and BAT after RvE1 treatment. In addition, we found that after treatment with RvE1, iWAT and BAT presented multiple thermogenesis fat features (Figs. 4h and S5k). These findings suggest that RvE1 is sufficient to orchestrate the hallmarks of thermogenesis in mice.

We then investigated the metabolic impact of RvE1 treatment. The glucose tolerance test (GTT) presented that RvE1 improved the glucose resistance of mice fed the HFD which showed no effect in similarly fed *Ucp1*^−/−^ mice (Fig. 4i). The insulin tolerance test (ITT) showed that RvE1 alleviated the insulin resistance of mice fed the HFD which showed no significance in *Ucp1*^−/−^ mice (Fig. 4j). Moreover, the fasting serum insulin level was reduced after RvE1 treatment (Fig. 4k) and the HOMA-IR also showed a robust improvement (Fig. 4l), which was dependent on UCP1. In addition, we measured oxygen consumption (Fig. 4m), carbon dioxide production (Fig. 4n), and calorie consumption (Fig. 4o) and found that RvE1 significantly augmented calorie-burning. Taken together, RvE1 induces the biogenesis of beige fat and improves the metabolic homeostasis in diet-induced obese mice against obesity and insulin resistance in vivo, which is UCP1 dependent.

### 3.5 RvE1 and chemerin control beige adipocyte biogenesis differentially via regulating mTORC1 signaling

**Fig. 5:**
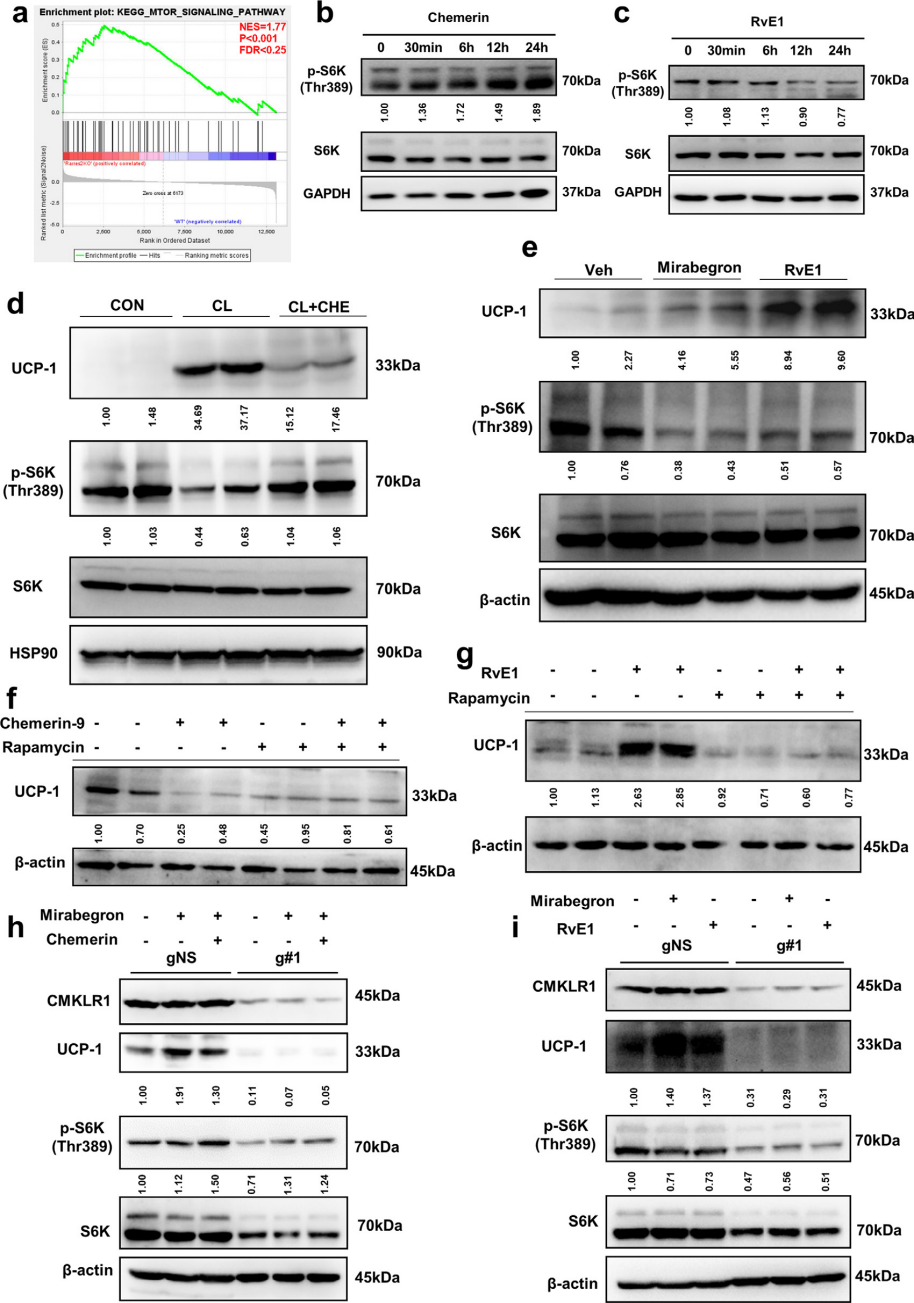
**Chemerin and RvE1 differentially regulated mTORC1 signaling via CMKLR1.** (a) Gene set enrichment analysis (GSEA) analysis for gene signatures of mTOR signaling pathway in iWAT from Rarres2 KO compared with C57BL/6J control mice. NES, normalized enrichment score. FDR, false discovery rate. (b, c) Western-blot analysis for level of p-S6K and S6K protein in undifferentiated 3T3-L1 treated with 100 ng/ml chemerin (b) or 100 ng/ml RvE1 (c). (d, e) Western-blot analysis for level of UCP1, p-S6K and S6K protein in 3T3-L1 mature adipocytes treated with 1 µM CL, 1 µM CL with 100 ng/ml chemerin (CL+CHE), 10 µM Mi or 100 ng/ml (0.285 µM) RvE1. (f) C57BL/6J mice fed with NCD for 8 weeks were injected with Vehicle, chemerin-9 (1 µg/mouse/day), Rapamycin (2 mg/kg/day) or Rapamycin (2 mg/kg/day) with chemerin-9 (1 µg/mouse/day) over 7 days. Western-blot analysis for level of UCP1 protein in iWAT. (g) C57BL/6J mice fed with NCD for 8 weeks were injected with Vehicle, RvE1 (1 µg/mouse/2day), Rapamycin (2 mg/kg/2day) or Rapamycin (2 mg/kg/2day) with RvE1 (1 µg/mouse/2day) over 14 days. Western-blot analysis for level of UCP1 protein in iWAT. (h-i) Western-blot analysis for level of p-S6K, S6K, UCP1 and CMKLR1 protein in different 3T3-L1 cell pools treated with 100 ng/ml chemerin, 10 µM Mirabegron or 100 ng/ml RvE1 as well as transfected with Cas9 and guide RNA targeting the *Cmklr1* gene sequence. gNS indicates 3T3-L1 cell pools transfected with Cas9 only and g#1 indicates 3T3-L1 cell pools transfected with Cas9 and guide RNA. The ImageJ software was used for gray scanning.

To elucidate the underlying mechanism by which RvE1 and chemerin regulate beige adipocyte development, GSEA was conducted to search the enriched KEGG pathways by group. For *Rarres2* knockout group, mTOR signaling pathway was the only gene enriched set based on the cut-off criteria of *p*-value < 0.05 and FDR < 0.25 (Fig. 5a). 3T3-L1 was treated with chemerin and RvE1, respectively, and proteins were collected at different time points and detected for the level of phosphorylated S6K (p-S6K), which reflects mTORC1 activity. Western-blot showed that chemerin treatment strongly promoted the phosphorylation of S6K (Fig. 5b), while RvE1 significantly inhibited the phosphorylation of S6K (Fig. 5c). Moreover, the level of UCP1 was increased and the level of p-S6K was decreased in 3T3-L1 mature adipocytes (Fig. 5d) and primary mature adipocytes (Fig. S6c) after being treated with β3-adrenoceptor agonist (CL or Mi), and these alternations were reversed by chemerin. Similarly, RvE1 treatment increased the level of UCP1 and decreased the level of p-S6K in 3T3-L1 mature adipocytes (Fig. 5e) and primary mature adipocytes (Fig. S6d).

To verify whether chemerin and RvE1 regulate the biogenesis of beige adipocyte via mTORC1 signaling pathway, we treated NCD-fed WT mice with chemerin-9 (the nonapeptide, 149-YFPGQFAFS-157) and rapamycin (inhibitor of mTORC1), respectively. Chemerin-9 was sufficient to inhibit the expression of UCP1 in iWAT (Fig. 5f) and BAT (Fig. S7a), which could be neutralized by rapamycin. Moreover, we found that RvE1 was sufficient to induce the expression of UCP1 in iWAT (Figs. 5g and S7b), which could be blocked by rapamycin. Taken together, these findings suggested that chemerin and RvE1 regulate the biogenesis of beige adipocytes dependent on the mTORC1 signaling pathway.

In addition, α-NETA (antagonist of CMKLR1) was used to verify whether the differential regulation effect of chemerin and RvE1 was dependent on CMKLR1. The results showed that α-NETA could significantly block the regulation of chemerin/RvE1 on mTORC1 signaling and UCP1 (Fig. S6e, 6f), indicating that the differential regulation effect of chemerin and RvE1 was dependent on CMKLR1. Furthermore, we found that CMKLR1 ablation by CRISPR–Cas9 with gRNAs targeting the CMKLR1 gene locus (Fig. S6g, 6h) significantly blocked the regulation effect of chemerin/RvE1 on mTORC1 signaling (Fig. 5h, 5i), which also suggests that chemerin and RvE1 regulate mTORC1 signaling via CMKLR1. In summary, chemerin and RvE1 control the biogenesis of beige adipocytes differentially via regulating CMKLR1-mTORC1 signaling.

### 3.6 RvE1 and chemerin exert differential regulation effect by forming hydrogen bonds with different binding sites of CMKLR1

**Fig. 6:**
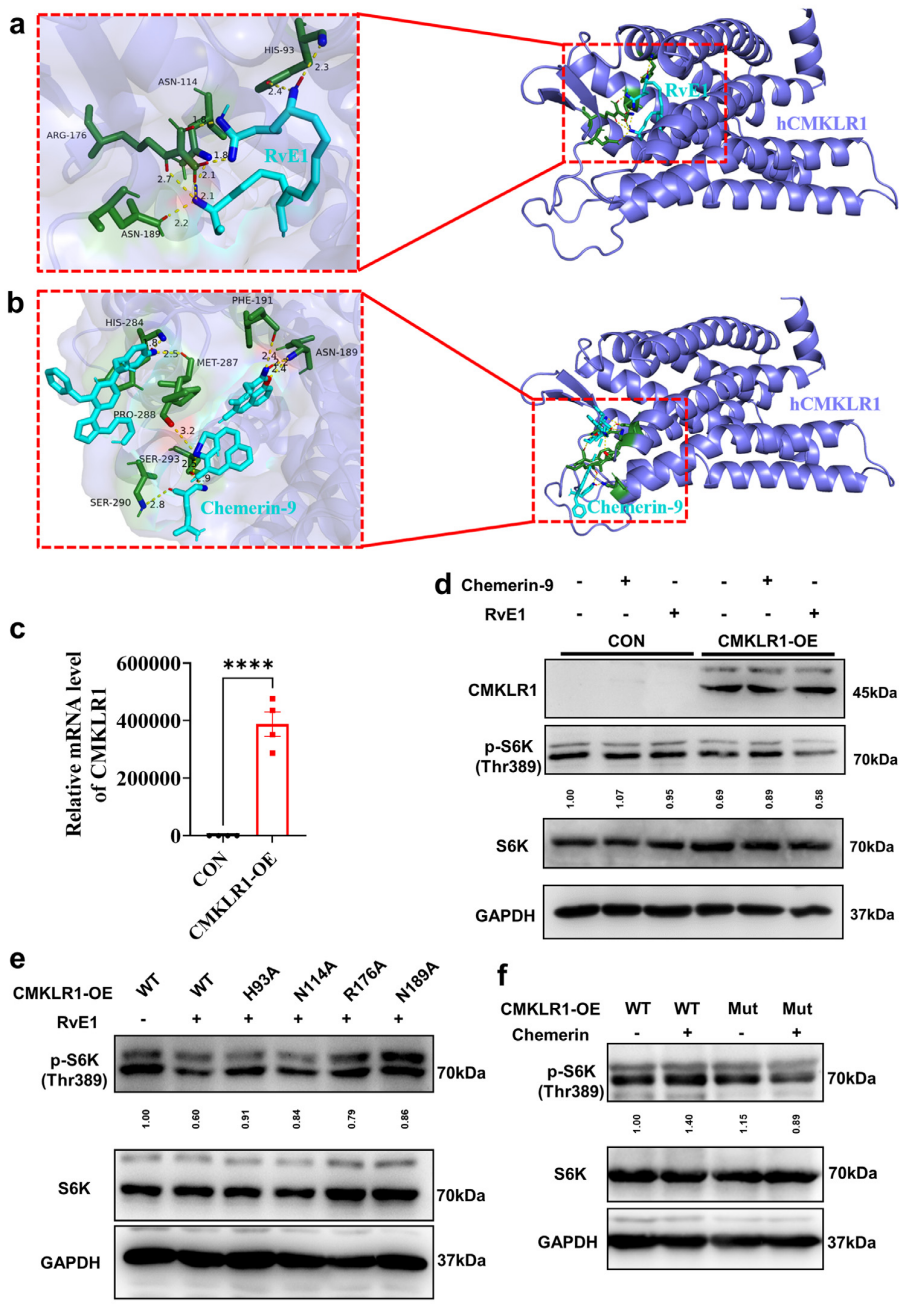
**RvE1 and chemerin exert differential regulatory effects by forming hydrogen bonds with different binding sites of CMKLR1.** (a, b) The binding modes of RvE1 (a) and chemerin-9 (b) to human CMKLR1 (hCMKLR1). hCMKLR1 is shown as a slate cartoon model, RvE1 and chemerin-9 are shown as a cyan sticks model, amino acid residues corresponding to each amino acid binding site are highlighted in forest and hydrogen bonds formed between the ligand and each amino acid residue are indicated by yellow dotted lines. The hydrogen bond distances are listed next to the red dotted line. Hydrogen bond donor atoms are shown in red and hydrogen bond acceptor atoms are shown in blue. (c) qPCR analysis of CMKLR1 in Hela. All data are presented as mean ± *SEM*. Statistical significance was determined by unpaired two-tailed Student's t-test. (d–f) Western-blot analysis for level of CMKLR1, p-S6K and S6K protein in Hela treated with 1 µM chemerin-9 or 100 ng/ml RvE1 as well as transfected with different plasmids of human CMKLR1. The ImageJ software was used for gray scanning.

To further explore how RvE1 and chemerin exert opposite regulatory effects on the mTORC1 signaling pathway through the same receptor CMKLR1, we predicted the binding sites of RvE1 and chemerin-9 to human CMKLR1 (hCMKLR1). The results showed that RvE1 formed hydrogen bonds with His-93, Asn-114, Arg-176 and Asn-189 of hCMKLR1 (Fig. 6a), while chemerin-9 formed hydrogen bonds with Asn-189, Phe-191, His-284, Met-287, Pro-288, Ser-290 and Ser-293 of hCMKLR1 (Fig. 6b), which indicated that RvE1 and chemerin-9 formed hydrogen bonds with different binding sites of hCMKLR1. In addition, we verified the reliability of the simulation model by predicting the binding site of α-NETA to hCMKLR1, which showed that α-NETA formed no hydrogen bond with hCMKLR1 (Fig. S7c) and its free binding energy (-28.576 kJ/mol) was lower than that of RvE1 (-22.342 kJ/mol) and chemerin-9 (-23.346 kJ/mol). These results suggest that our computational models are practical. At the same time, the amino acid sequence alignment of CMKLR1 showed that the binding sites of RvE1 and chemerin-9 to CMKLR1 were almost all located in the common amino acid residues of human and mouse (Fig. S7d), indicating that the binding sites of RvE1 and chemerin-9 to CMKLR1 may be conservative. Therefore, the binding sites of α-NETA, RvE1 and chemerin-9 to mouse CMKLR1 (mCMKLR1) were predicted, which showed that α-NETA formed no hydrogen bond with mCMKLR1 (Fig. S8a) and its free binding energy (-27.949 kJ/mol) was lower than that of RvE1 (-24.058 kJ/mol) and chemerin-9 (-25.732 kJ/mol). Surprisingly, the result showed that RvE1 formed hydrogen bonds with Asp-18, Lys-107, Lys-111, Val-174, Asn-190, Ala-194 and Glu-197 of mCMKLR1 (Fig. S8b), which was different from the predicted result of RvE1 to hCMKLR1 (Fig. 6a). Moreover, the binding sites of chemerin-9 to mCMKLR1 are Asp-25, Asn-189 and Ser-293 (Fig. S8c), which were partially consistent with the binding sites of chemerin-9 to hCMKLR1 (Fig. 6b). However, although the predicted binding sites of RvE1 are different in hCMKLR1 and mCMKLR1, these results showed that RvE1 and chemerin-9 may form hydrogen bonds with different binding sites of mCMKLR1 (Fig. S8b, S8c).

Then, we verified whether the differential regulatory effect of chemerin and RvE1 on mTORC1 also exists in human CMKLR1 by overexpressing human CMKLR1 in a Hela cell line which has low expression of CMKLR1 (Fig. 6c). We found that RvE1 and chemerin-9 could not regulate mTORC1 signaling in Hela, but RvE1 inhibited mTORC1 signaling and chemerin-9 activated mTORC1 (Fig. 6d) signaling in Hela which overexpressed human CMKLR1. Furthermore, the four amino acid residues His-93, Asn-114, Arg-176 and Asn-189 of human CMKLR1 were mutated into alanine, respectively, to verify whether RvE1 inhibits the mTORC1 signaling pathway depended on these amino acid residues. The results showed that mutations at these four amino acid residues did not affect basal level of mTORC1 signaling (Fig. S8d), but significantly prevented RvE1 from inhibiting mTORC1 signaling (Fig. 6e). Moreover, the seven amino acid residues Asn-189, Phe-191, His-284, Met-287, Pro-288, Ser-290 and Ser-293 of human CMKLR1 were mutated into alanine to verify whether chemerin-9 activates the mTORC1 signaling pathway depended on these amino acid residues. The results showed that mutations at these seven amino acid residues significantly prevented chemerin-9 from activating mTORC1 signaling (Fig. 6f). These findings suggest that RvE1 and chemerin may exert a differential regulation effect by forming hydrogen bonds with different binding sites of hCMKLR1 (Fig. S8e).

## 4 Discussion

In this study we have demonstrated a novel function of chemerin and RvE1 acting via CMKLR1 in modulating the development of beige fat differentially. RvE1 and chemerin regulated the biogenesis of beige fat in WAT and played a role in systemic glucose homeostasis. RvE1 induced the beige fat to activate the adipose thermogenic program and facilitate metabolic homeostasis, whereas chemerin inhibited the beige fat development thereby suppressing thermogenesis and impairing metabolic homeostasis. Moreover, both RvE1 and chemerin function through the mTORC1 signaling pathway. The novelty of this current study lies in the finding that RvE1 and Chemerin regulate the progress of beige fat and influence metabolic homeostasis subsequently, implying that the Chemerin/RvE1-CMKLR1-mTORC1 signaling pathway may reveal a potential therapeutic target against obesity and the related metabolic disorders.

Induction of beige fat has been explored as a promising therapeutic strategy against obesity [28]. While the β3-adrenoceptor agonist effectively promoted thermogenesis in brown and beige adipocytes in rodents, the clinical implications seem unfeasible due to the low efficacy of β3-adrenoceptor agonists in humans [29,30]. It is crucial to explore other potential therapeutic targets that can specifically and efficiently promote beige adipogenesis against obesity and the associated metabolic disorders. Here we identified CMKLR1 as a novel GPCR therapeutic target that can regulate the biogenesis of beige fat by its two contrary conjugates, chemerin and RvE1. In previous reports, mice deficient in CMKLR1 showed attenuated androgen-induced lipid accumulation, which suggests an anti-obesity role of CMKLR1 by regulating lipid accumulation [31]. Moreover, a previous study showed that the lack of chemerin and adipocytic CMKLR1 could enhance cold-induced thermogenic beige fat via potentiating the type 2 innate immune responses, suggesting that the chemerin-CMKLR1 axis is a physiological negative regulator of thermogenic beige fat [17]. Nevertheless, the effects of chemerin treatment on the biogenesis of beige adipocytes and the RvE1-CMKLR1 axis on beige fat have not been explored. Previous studies showed that chemerin exacerbated insulin resistance [16,32] and was involved in differentiation of adipocytes [14,33], but whether chemerin affects metabolism by regulating the biogenesis of the beige adipose tissue was uncertain. Consequently, we confirmed that chemerin deteriorates obesity and impairs insulin sensitivity through inhibiting the thermogenesis program of adipose tissue.

The association of RvE1 with obesity and its associated metabolic diseases, such as type 2 diabetes, was thought to be mediated by RvE1 or its precursor omega-3-PUFAs increasing adiponectin [34,35], but whether and how RvE1 is directly involved in metabolic homeostasis is unknown. Our study showed that RvE1 reduces body weight and improves metabolic homeostasis by inducing the thermogenesis program of adipose tissue. Moreover, we found that chemerin and RvE1 also significantly affected the thermogenic program of BAT, although CMKLR1 was screened out because it was highly expressed in WAT compared with BAT. We suggest that this may be due to the moderate expression of CMKLR1 in BAT compared to the high expression of CMKLR1 in WAT and low expression of CMKLR1 in the liver (Figs. S1e and S2h).

The mechanistic targets of rapamycin (mTOR) have two different complexes, mTORC1 and mTORC2, that differ in both subunit compositions and biological function [36]. mTORC1 was reported to regulate various metabolic processes such as lipogenesis, protein synthesis, energy expenditure, and autophagy and it was highly activated in the fat of obese and HFD-fed rodents [37,38]. Nevertheless, its roles in the biogenesis of beige adipocytes are in question [39–41]. Some studies showed that inhibition of mTORC1 resulted in the beiging of WAT and that activation of mTORC1 suppressed thermogenesis in adipose tissue, which exacerbated obesity and insulin resistance in mice [40,41]. Generally, mTORC1 is a potent regulator of beige adipogenesis and mTORC1 signaling inhibition in adipose tissue under the obese state is conducive to the biogenesis of beige fat [41]. However, other studies showed that loss of mTORC1 signaling in adipose tissue increased the expression of thermogenic genes under normal physiological conditions, but impaired adipose thermogenesis induced by β-AR agonists and cold stimulation [42,43]. These studies imply that mTORC1 may play distinct roles in adipose thermogenesis under different conditions. Moreover, our present study shows that RvE1 activates adipose thermogenesis by inhibiting mTORC1 signaling, but inhibiting mTORC1 by rapamycin does not exert a similar effect (Figs. 5g and S7b). These seemingly inconsistent results may be due to that RvE1 inhibits mTORC1 signaling specifically in tissues expressing CMKLR1 (such as WAT) while rapamycin broadly suppresses the mTORC1 signaling. Therefore, comparing with the specific inhibition of mTORC1 in CMKLR1-expressed tissues by RvE1, a systemic inhibition of mTORC1 by rapamycin may lead to different effects on an adipose thermogenic program through organ crosstalk. Consistent with those results, previous reports showed that rapamycin treatment did not promote adipose thermogenesis, while adipocyte-specific ablation of mTORC1 increased the basal expression of the thermogenic genes [41,43]. In summary, we suggested that the regulation of the CMKLR1-mTORC1 axis is critically involved in beige biogenesis regulated by RvE1 and chemerin in this study.

We chose not to focus on the regulatory role of RvE1/Chemerin-CMKLR1 signaling in inflammation, which is well established [[44], [45], [46], [47]]. Given its high expression across white fat depots in both mice and humans, further studies are needed to determine whether CMKLR1 performs a similar role in humans. Nevertheless, our findings underlie a potential therapeutic feature of CMKLR1 in obesity and the associated metabolic diseases from the thermogenic viewpoint of beige fat.

## 5 Conclusion

In conclusion, opposite to the function of chemerin, RvE1 promoted lipid metabolism and energy production, induced the biogenesis of beige adipocytes, and alleviated lipid accumulation in the adipose tissues via suppressing the mTORC1 signaling. Given the etiological significance of identifying signaling pathways that induce beige fat and restore obesity-linked dysfunction in adipose tissue, our findings indicate that modulation of the intracellular signaling of CMKLR1 may be a potentially novel therapeutic strategy.

## Ethical approval

All the animal experiments were conducted with the approval of the Animal Care and Use Committee of Sun Yat-sen University (Approval ID: SCXK2011-0029). The sample collection from patients was approved by the Medical Ethics Committee of Zhongshan School of Medicine (Approval ID: ZSSOM-MEC [2017] #045). This study was conducted in accordance with the ethical principles derived from the Declaration of Helsinki and Belmont Report and was approved by the review board of Sun Yat-sen University (Guangzhou, China).

## Author contributions

**Zewei Zhao:** Investigation, Conceptualization, Data curation, Formal analysis, Writing – original draft. **Siqi Liu:** Data curation, Investigation. **Bingxiu Qian:** Data curation, Investigation. **Lin Zhou:** Data curation, Formal analysis. **Jianglin Shi:** Data curation, Formal analysis. **Junxi Liu:** Data curation, Formal analysis. **Lin Xu:** Writing – review & editing. **Zhonghan Yang:** Funding acquisition, Conceptualization, Supervision, Writing – review & editing.

## Declaration of competing interest

The authors declare that they have no conflicts of interest in this work.

## Appendix Supplementary materials

Image, application 1
