# Supplementary material for: CMKLR1 senses chemerin/resolvin E1 to control adipose thermogenesis and modulate metabolic homeostasis
Source: Fundam Res. 2022 Jul 4;4(3):575–88. doi: 10.1016/j.fmre.2022.06.014 (PMC11197767; doi:10.1016/j.fmre.2022.06.014)
Supplement: Supplementary file 1 [file mmc1.docx]

**Supplementary figure**

**
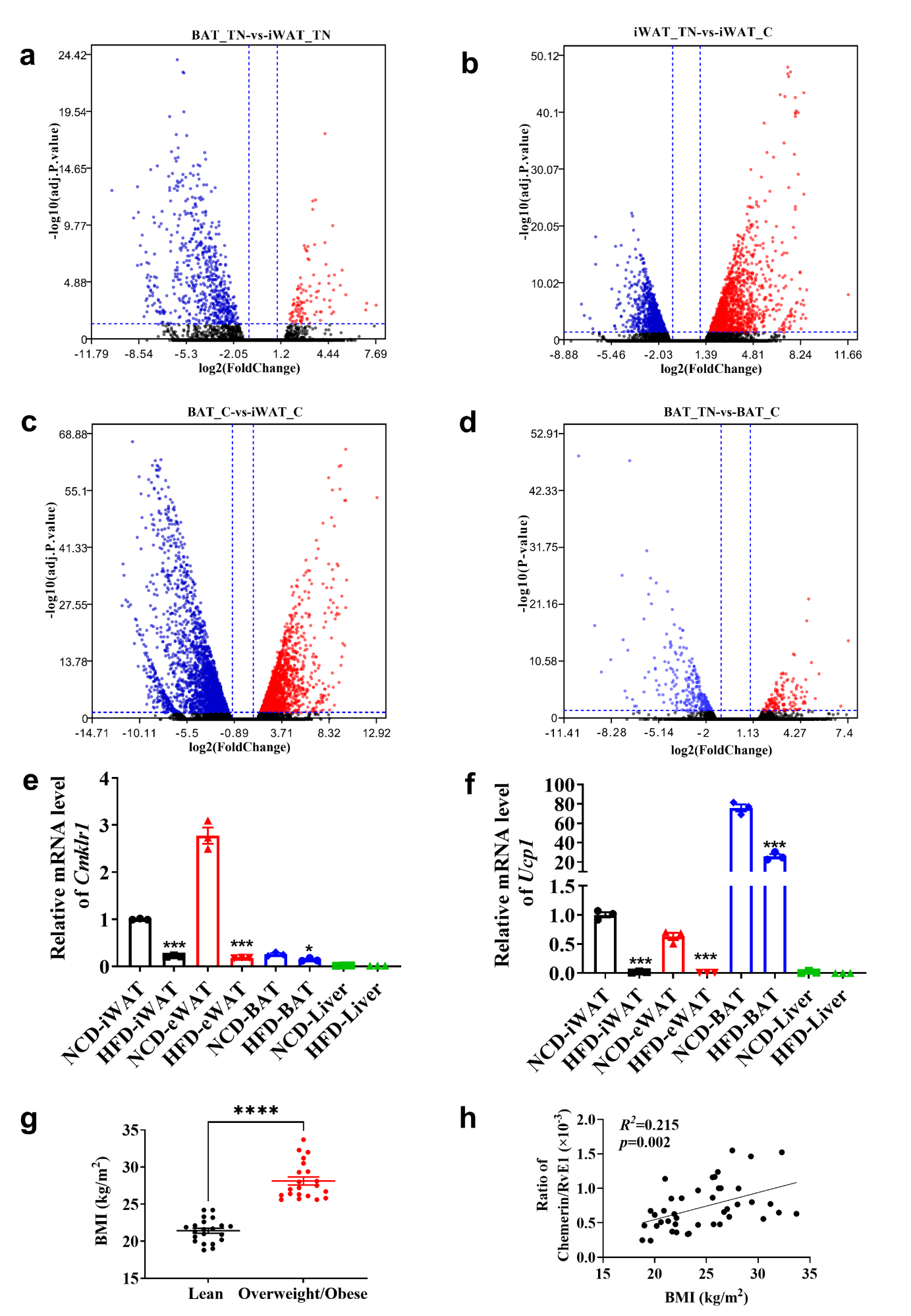
**

**Fig. S1. G-protein coupled receptor screening and CMKLR1 was down-regulated in mice with DIO.** (a-d) Volcano plot summarizing the differentially expressed genes (DEGs) between thermal-neutral iWAT group and thermal-neutral BAT group (a), the DEGs between thermal-neutral iWAT group and cold temperature iWAT group (b), the DEGs between cold temperature iWAT group and cold temperature BAT group (c) and the differentially expressed genes between thermal-neutral BAT group and cold temperature BAT group (d). Blue and red shading indicate down-regulation and up-regulation, respectively. (e-f) qPCR analysis of *Cmklr1* and *Ucp1* in different tissue from mice fed with normal chow diet (NCD) or high fat diet (HFD) for 12 weeks. (g) The body mass index (BMI) of lean and overweight/obese human. Lean (BMI = 18.5~23.9) n = 21, Overweight/Obese (BMI > 25.5) n = 21. (h) Correlation between ratio of (serum chemerin level) / (serum RvE1 level) and body mass index (BMI). All data are presented as mean ± *SEM*. Statistical significance was determined by unpaired two-tailed Student’s t-test (e-g) or simple linear regression (h).

**
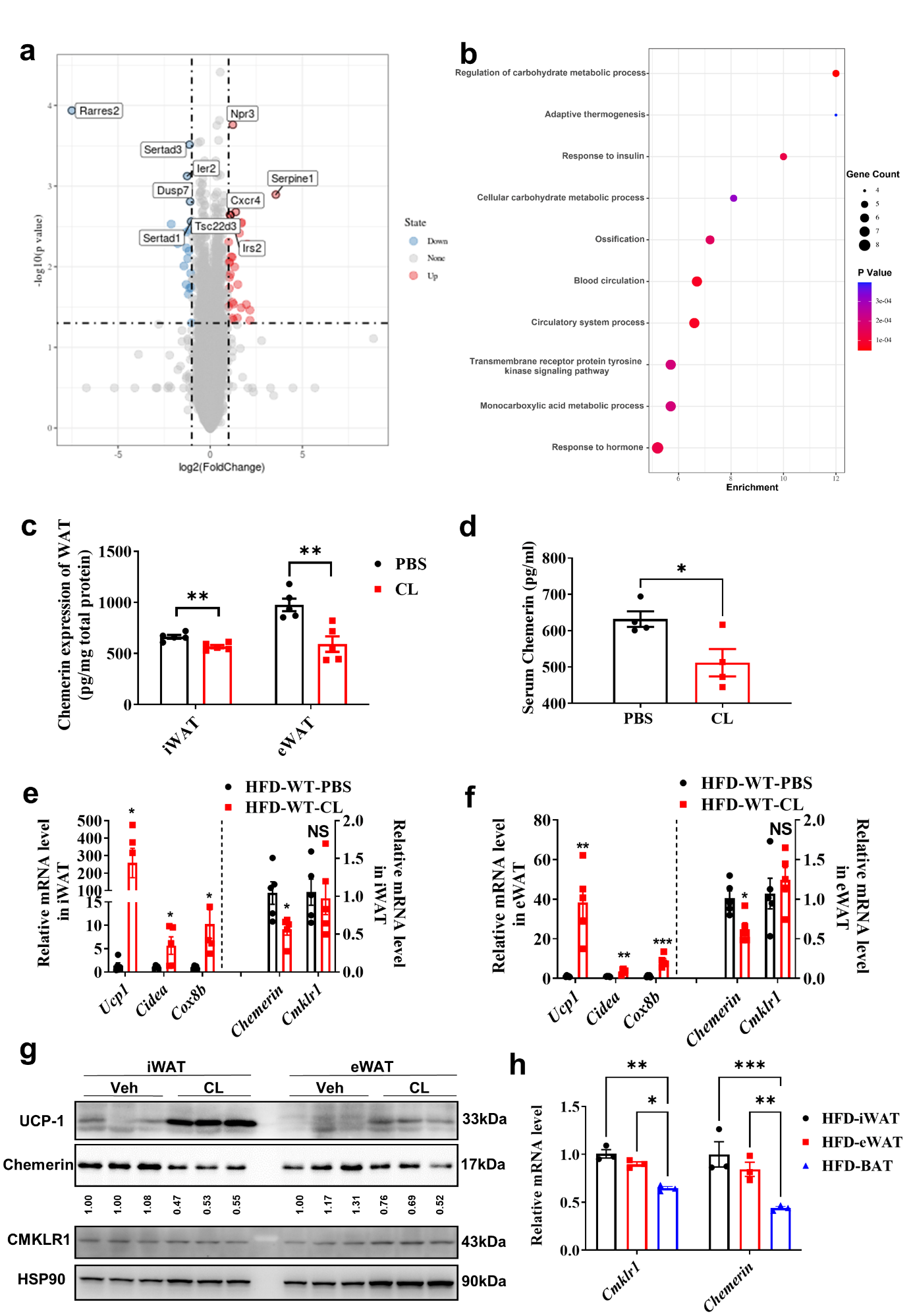
**

**Fig. S2. Chemerin negatively correlated with beige fat.** RNA-sequencing gene expression signatures of iWAT from 8-week-old male *Rarres2* KO and C57BL/6J control mice. n = 3/group. (a) Volcano plot summarizing differential gene expression in *Rarres2* knockout inguinal white adipose tissue (iWAT) versus control samples and illustrating gene expression signatures for *Rarres2* knockout iWAT. Blue and red shading indicate down-regulation and up-regulation, respectively, as shown in the color key at the right. (b) Gene Ontology (GO) enrichment analysis for the differential genes. The color represents the adjusted p-values (q-value), and the size of the spots represents the gene number. (c-f) The level of chemerin in the iWAT, eWAT (c, e, f) and serum (d) of mice treated with vehicle or CL (1mg/kg/day) over 7 days. The level of chemerin was measured by an ELISA and qPCR in the iWAT, eWAT and serum. (g) The expression of UCP1, chemerin and CMKLR1 in the iWAT and eWAT of mice treated with vehicle or CL (1mg/kg/day) over 7 days. The ImageJ software was used for gray scanning. (h) The relative mRNA level of *Chemerin* and *Cmklr1* in the iWAT, eWAT and BAT of HFD-fed mice. iWAT, inguinal white adipose tissue; eWAT, epididymal white adipose tissue; HFD, high-fat diet; RvE1, Resolvin E1; NCD, normal chow diet; qPCR, quantitative real-time PCR. All data are presented as mean ± *SEM*. Statistical significance was determined by unpaired two-tailed Student’s t-test (c-f) and one-way ANOVA (h).


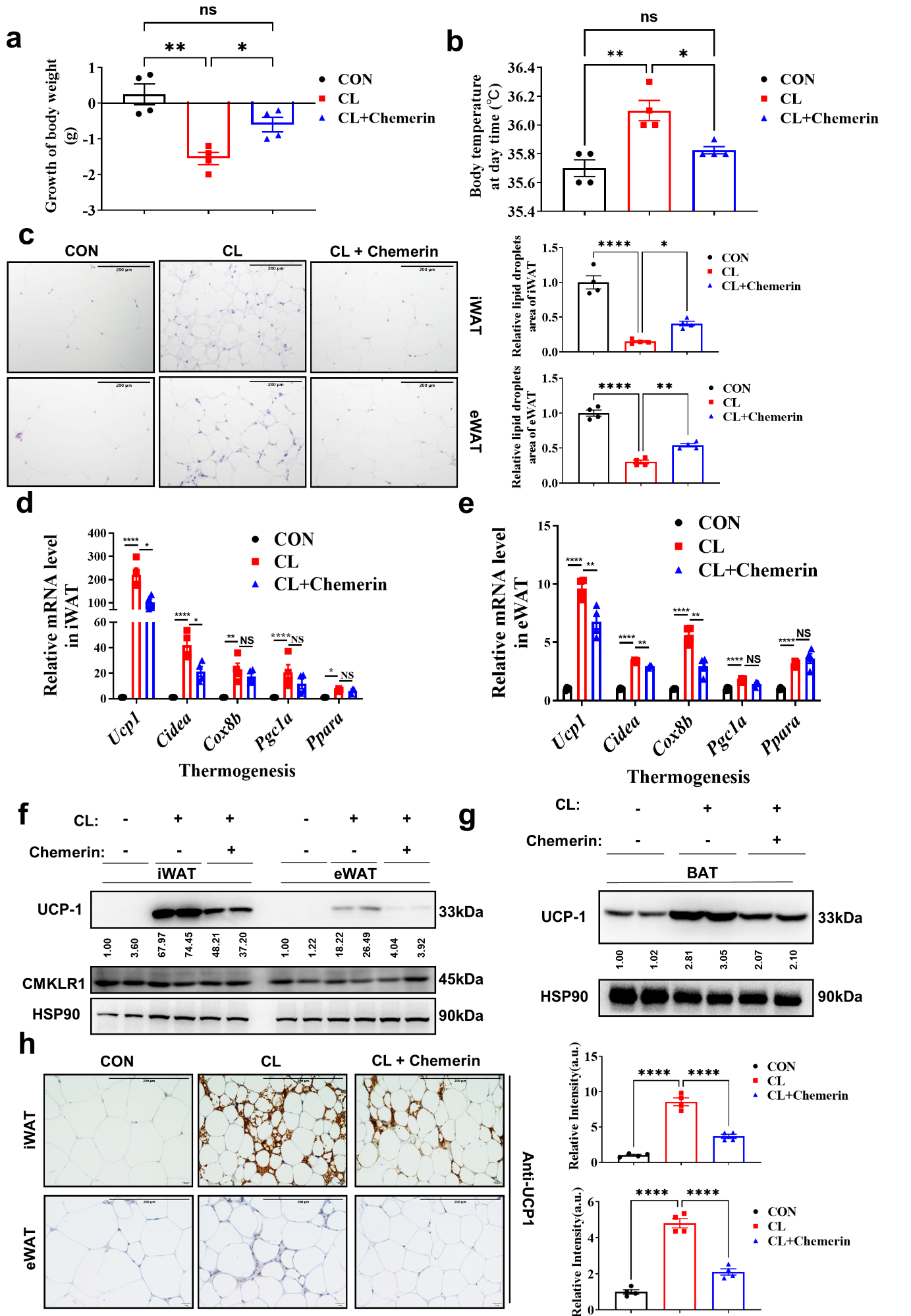


**Fig. S3. Chemerin inhibited the biogenesis of beige fat induced by β3-AR agonist in mice with DIO.** (a-h) C57BL/6J mice fed with HFD for 12 weeks (n = 4 per treatment) were injected with vehicle, CL (1mg/kg/2day) or CL (1mg/kg/2day) + chemerin (10ng/g/day) over 5 days. (a-b) Changes in body mass (a) and body temperature (b) in HFD induced obese mice were treated differently. (c) Representative images of iWAT (top) and eWAT (bottom) stained with HE. Adipocyte size was analyzed using the ImageJ software. Scale bars, 200 μm. (d-e) qPCR analysis of markers associated with the biogenesis of beige fat and thermogenesis in iWAT (d) and eWAT (e) from different treatment mice. (f-g) Western-blot analysis for level of UCP1 protein in iWAT (f-left), eWAT (f-right) and BAT (g) of HFD mice treated with vehicle, CL (1mg/kg/2day) or CL (1mg/kg/2day) + chemerin (10ng/g/day) over 5 days. The ImageJ software was used for gray scanning. (h) Representative images of iWAT (top) and eWAT (bottom) stained with UCP1. Scale bars, 200 μm. The ImageJ software was used for staining intensity analysis. All data are presented as mean ± *SEM*. Statistical significance was determined by one-way ANOVA (a-e, h).

 
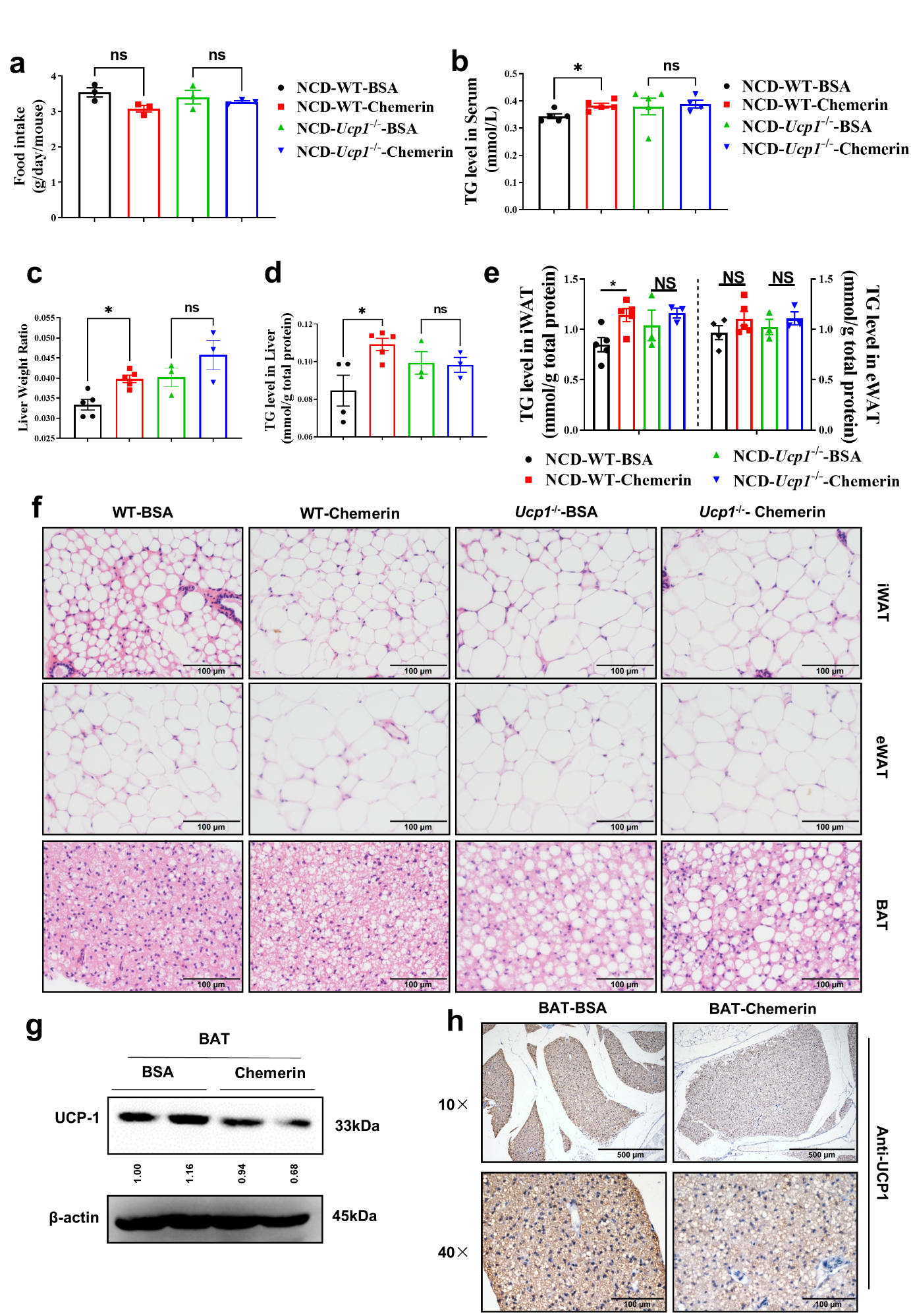


**Fig. S4. Characterization of wild-type and *Ucp1*-knockout mice after chemerin treatment.** (a-h) C57BL/6J mice or *Ucp1* knockout mice fed with NCD for 8 weeks (n = 5 per treatment) were injected with BSA (10ng/g/day) or chemerin (10ng/g/day) over 14 days. (a, c) Food intake (a) and liver weight ratio (c) of different treated mice. (b, d-e) The TG level of serum(b), liver (d), iWAT (e-left) and eWAT (e-right) from different treated mice. (f) Representative images of iWAT (top), eWAT (middle) and BAT (bottom) stained with hematoxylin and eosin. Scale bars, 100 μm. (g) Western-blot analysis for level of UCP1 protein in BAT from different treated mice. The ImageJ software was used for gray scanning. (h) Representative images of BAT stained with UCP1. Scale bars, 100 μm. All data are presented as mean ± *SEM*. Statistical significance was determined by one-way ANOVA (a-e).


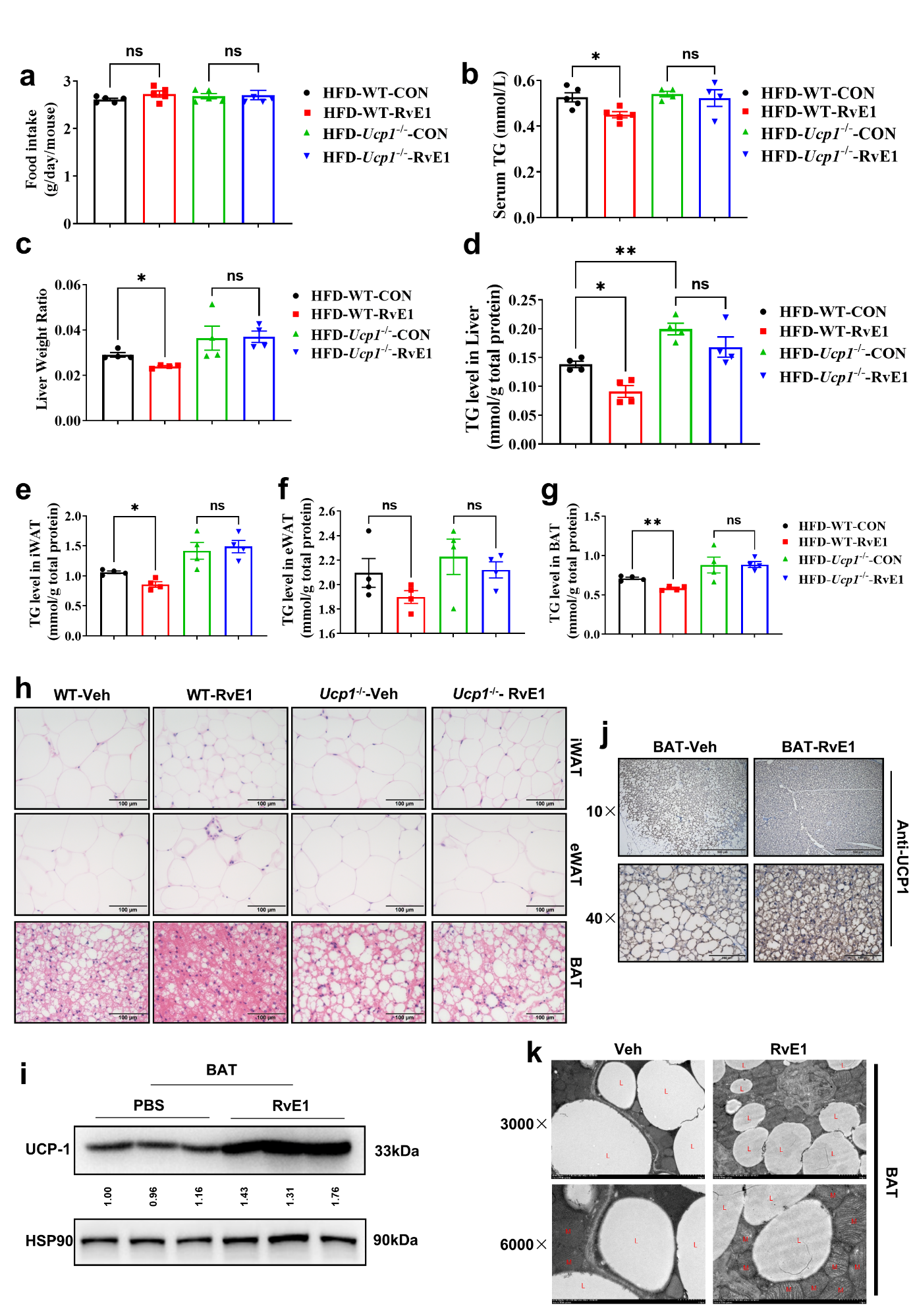


**Fig. S5. Characterization of C57BL/6J wild-type and *Ucp1*-knockout mice after RvE1 treatment.** (a-k) C57BL/6J mice fed with HFD for 12 weeks (n = 5 per treatment) were injected with vehicle or RvE1 (1μg/mouse) over 14 days. (a, c) Food intake (a) and liver weight ratio (c) of different treated mice. (b, d-g) The TG level of serum (b), liver (d), iWAT (e), eWAT (f) and BAT (g) from different treated mice. (h) Representative images of iWAT (top), eWAT (middle) and BAT (bottom) stained with hematoxylin and eosin. Scale bars, 100 μm. (i) Western-blot analysis for level of UCP1 protein in BAT from different treated mice. The ImageJ software was used for gray scanning. (j) Representative images of BAT stained with UCP1. Scale bars, 100 μm. (k) Transmission electron microscope photograph of BAT treated with vehicle or RvE1. The red ‘L’ marks represent lipid droplets and the red ‘M’ marks represent mitochondria. All data are presented as mean ± *SEM*. Statistical significance was determined by one-way ANOVA (a-g).


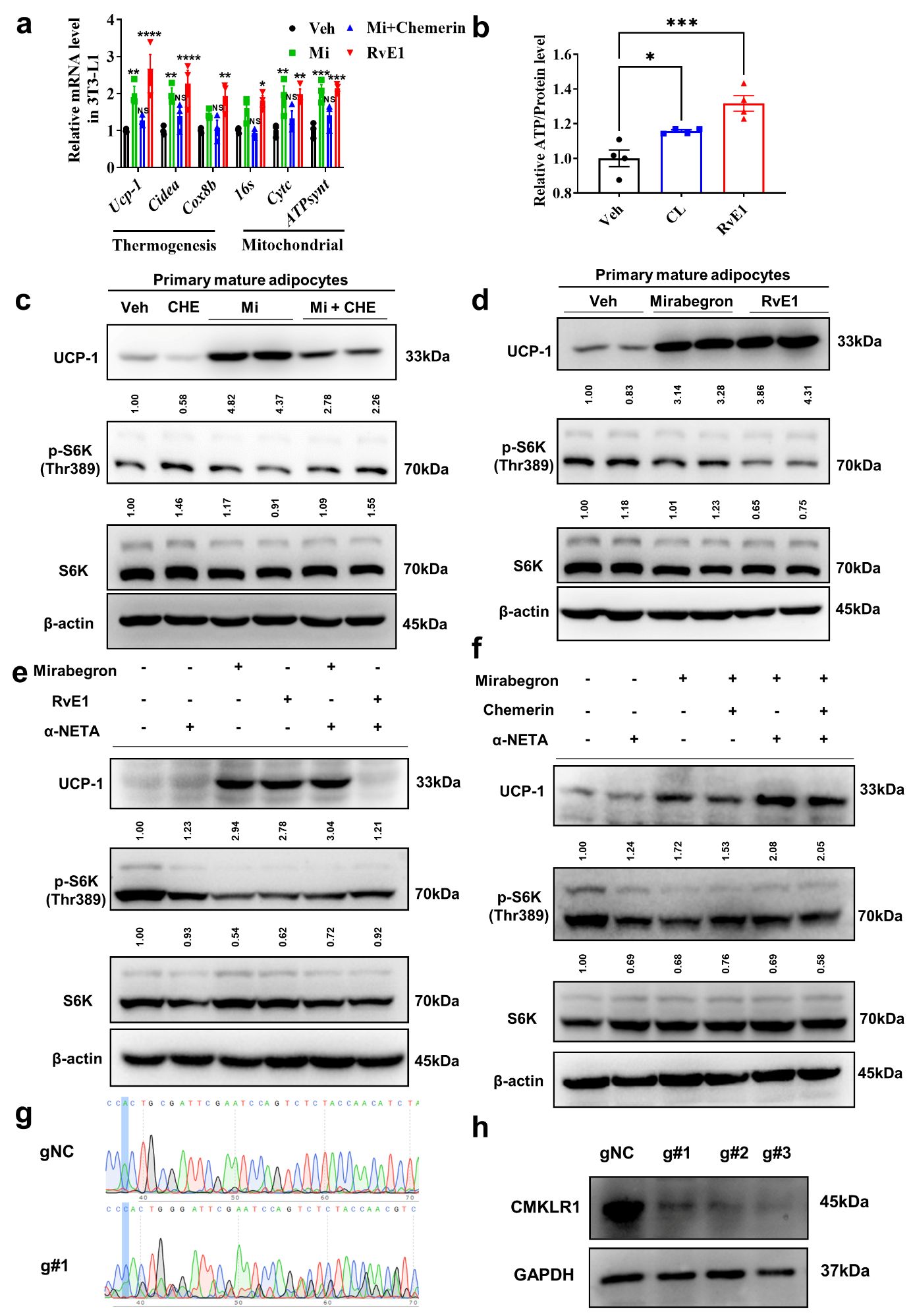


**Fig. S6. CMKLR1 was responsible for the differential regulation of RvE1 and chemerin on the biogenesis of beige adipocytes.** (a) qPCR analysis of markers associated with biogenesis of beige adipocytes in 3T3-L1 mature adipocytes. (b) ATP production in 3T3-L1 cells treated with vehicle or CL (1 μM) or RvE1 (100 ng/ml). (c-d) Western-blot analysis for level of UCP1, p-S6K and S6K protein in primary mature adipocytes treated with vehicle (Veh), 100 ng/ml chemerin (CHE), 10 μM Mirabegron (Mi), 10 μM Mi with 100 ng/ml chemerin (Mi+CHE) or 100 ng/ml RvE1. (e-f) Western-blot analysis for level of UCP1, p-S6K and S6K protein in 3T3-L1 mature adipocytes treated with 10 μM Mi, 100 ng/ml RvE1,100 ng/ml chemerin or 5 μM α-NETA. (g) The Sanger sequencing analysis of genomic DNA from 3T3-L1 cells transduced by lentivirus encoding gRNAs targeting CMKLR1, along with gNS. Blue highlights indicate the start sites of the disrupted genome sequence. (h) Western-blot analysis for the level of CMKLR1 protein in different 3T3-L1 cell pools transfected with Cas9 and guide RNA targeting the *Cmklr1* gene sequence. gNS indicates 3T3-L1 cell pools transfected with Cas9 only, g#1, g#2 and g#3 indicate different 3T3-L1 cell pools transfected with Cas9 and guide RNA. All data are presented as mean ± *SEM*. Statistical significance was determined by one-way ANOVA (a, d). The ImageJ software was used for gray scanning.


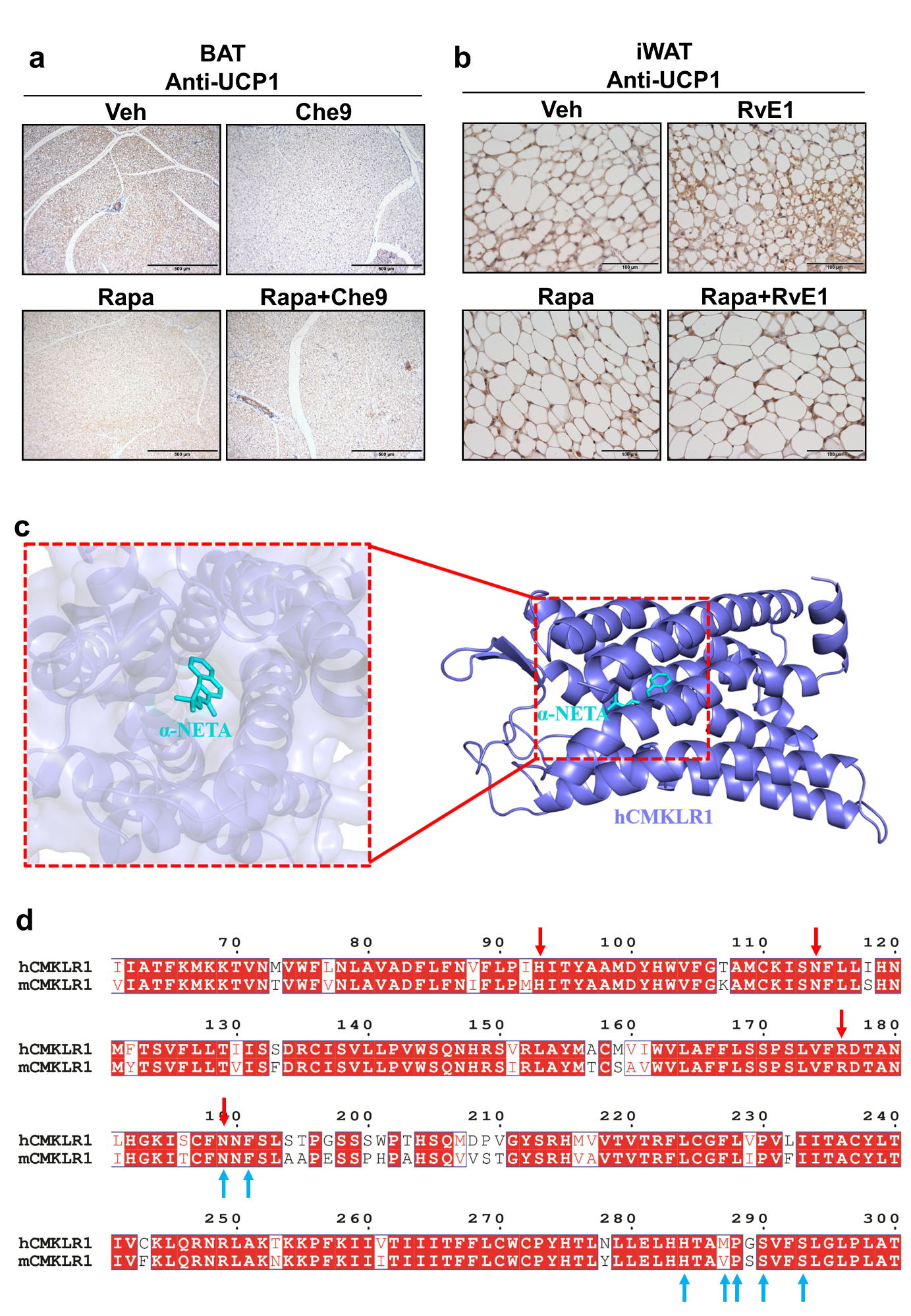


**Fig. S7. The differential regulatory effect of chemerin and RvE1 on beige fat depends on mTORC1.** (a) C57BL/6J mice fed with NCD for 8 weeks were injected with Vehicle, chemerin-9 (1μg/mouse/day), Rapamycin (2mg/kg/day) or Rapamycin (2mg/kg/day) with chemerin-9 (1μg/mouse/day) over 7 days. Representative images of BAT stained with UCP1. Scale bars, 500 μm. (b) C57BL/6J mice fed with NCD for 8 weeks were injected with Vehicle, RvE1 (1μg/mouse/2day), Rapamycin (2mg/kg/2day) or Rapamycin (2mg/kg/2day) with RvE1 (1μg/mouse/2day) over 14 days. Representative images of iWAT stained with UCP1. Scale bars, 100 μm. (c) The binding modes of α-NETA to hCMKLR1. hCMKLR1 is shown as a slate cartoon model and α-NETA is shown as a cyan sticks model. (d) Amino acid sequence alignment map of CMKLR1 in human and mouse. The red arrow points to the RvE1 amino acid binding site on hCMKLR1 and the blue arrow points to the chemerin-9 amino acid binding site on hCMKLR1.


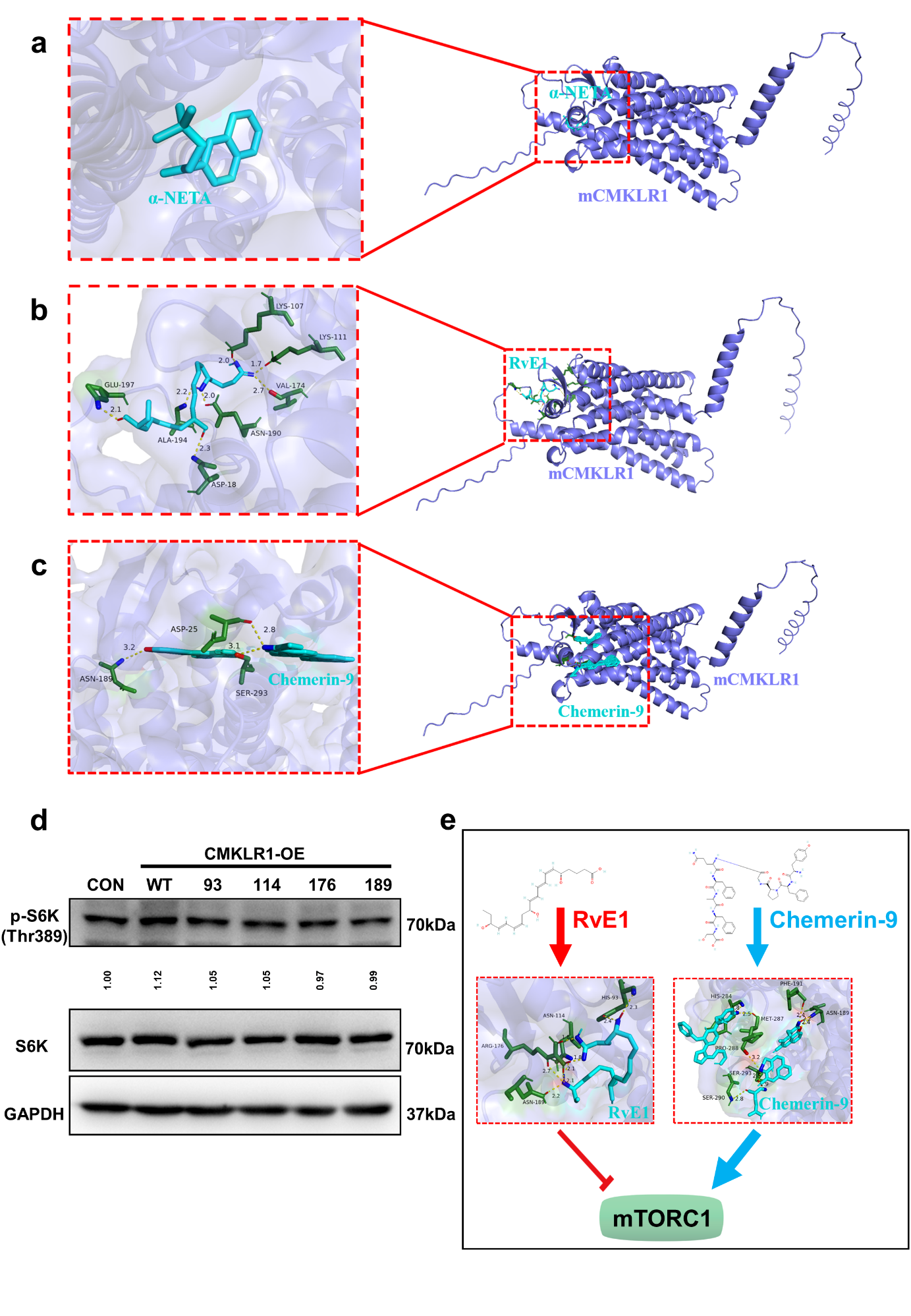


**Fig. S8. The binding site of chemerin-9 to CMKLR1 is different from that of RvE1.** (a-c) The binding modes of α-NETA (a), RvE1 (b) and chemerin-9 (c) to mouse CMKLR1 (mCMKLR1). mCMKLR1 is shown as a slate cartoon model, α-NETA, RvE1 and chemerin-9 are shown as a cyan sticks model, amino acid residues corresponding to each amino acid binding site are highlighted in forest and hydrogen bonds formed between the ligand and each amino acid residue are indicated by yellow dotted lines. The hydrogen bond distances are listed next to the red dotted line. Hydrogen bond donor atoms are shown in red and hydrogen bond acceptor atoms are shown in blue. (d) Western-blot analysis for level of p-S6K and S6K protein in Hela transfected with different plasmids of human CMKLR1. The ImageJ software was used for gray scanning. (e) Working model of RvE1 and chemerin-9 exert differential regulation effect by forming hydrogen bonds with different binding sites of hCMKLR1.
